# High Lipid Content of Prey Fish and *n*−*3* PUFA Peroxidation Impair the Thiamine Status of Feeding-Migrating Atlantic Salmon (*Salmo salar*) and Is Reflected in Hepatic Biochemical Indices

**DOI:** 10.3390/biom12040526

**Published:** 2022-03-30

**Authors:** Marja Keinänen, Soili Nikonen, Reijo Käkelä, Tiina Ritvanen, Mervi Rokka, Timo Myllylä, Jukka Pönni, Pekka J. Vuorinen

**Affiliations:** 1 Natural Resources, Fisheries and Fish Resources, Natural Resources Institute Finland (Luke), Latokartanonkaari 9, FI-00790 Helsinki, Finland; m.e.keinanen@gmail.com (M.K.); jukka.ponni@luke.fi (J.P.); 2 Chemistry Unit, Laboratory and Research Division, Finnish Food Authority, Mustialankatu 3, FI-00790 Helsinki, Finland; soili.nikonen@ruokavirasto.fi (S.N.); tiina.ritvanen@ruokavirasto.fi (T.R.); mervi.rokka@ruokavirasto.fi (M.R.); 3 Helsinki University Lipidomics Unit (HiLIPID), Helsinki Institute of Life Science (HiLIFE) and Biocenter Finland, University of Helsinki, Viikinkaari 1, FI-00014 Helsinki, Finland; reijo.kakela@helsinki.fi; 4 Molecular and Integrative Biosciences Research Programme, Faculty of Biological and Environmental Sciences, University of Helsinki, Viikinkaari 1, FI-00014 Helsinki, Finland; 5 Natural Resources, Fisheries and Fish Resources, Natural Resources Institute Finland (Luke), Itäinen Pitkäkatu 4 A, FI-20520 Turku, Finland; timo.myllyla@luke.fi; 6 Faculty of Biological and Environmental Sciences, University of Helsinki, Viikinkaari 1, FI-00014 Helsinki, Finland; 7 Department of Biological and Environmental Science, University of Jyväskylä, Survontie 9 C, FI-40014 Jyväskylä, Finland

**Keywords:** Atlantic salmon *Salmo salar*, Baltic Sea, herring *Clupea harengus*, lipid peroxidation, M74 syndrome, malondialdehyde, polyunsaturated fatty acids, sprat *Sprattus sprattus*, thiamine, total lipids

## Abstract

Signs of impaired thiamine (vitamin B1) status in feeding-migrating Atlantic salmon (*Salmo salar*) were studied in three Baltic Sea areas, which differ in the proportion and nutritional composition of prey fish sprat (*Sprattus sprattus*) and herring *(Clupea harengus)*. The concentration of *n*−*3* polyunsaturated fatty acids (*n*−*3* PUFAs) increased in salmon with dietary lipids and *n*−*3* PUFAs, and the hepatic peroxidation product malondialdehyde (MDA) concentration increased exponentially with increasing *n*−*3* PUFA and docosahexaenoic acid (DHA, 22:6*n*−*3*) concentration, whereas hepatic total thiamine concentration, a sensitive indicator of thiamine status, decreased with the increase in both body lipid and *n*−*3* PUFA or DHA concentration. The hepatic glucose 6-phosphate dehydrogenase activity was suppressed by high dietary lipids. In salmon muscle and in prey fish, the proportion of thiamine pyrophosphate increased, and that of free thiamine decreased, with increasing body lipid content or PUFAs, or merely DHA. The thiamine status of salmon was impaired mainly due to the peroxidation of *n*−*3* PUFAs, whereas lipids as a source of metabolic energy had less effect. Organochlorines or general oxidative stress did not affect the thiamine status. The amount of lipids, and, specifically, their long-chain *n*−*3* PUFAs, are thus responsible for generating thiamine deficiency, and not a prey fish species per se.

## 1. Introduction

**Figure 1:**
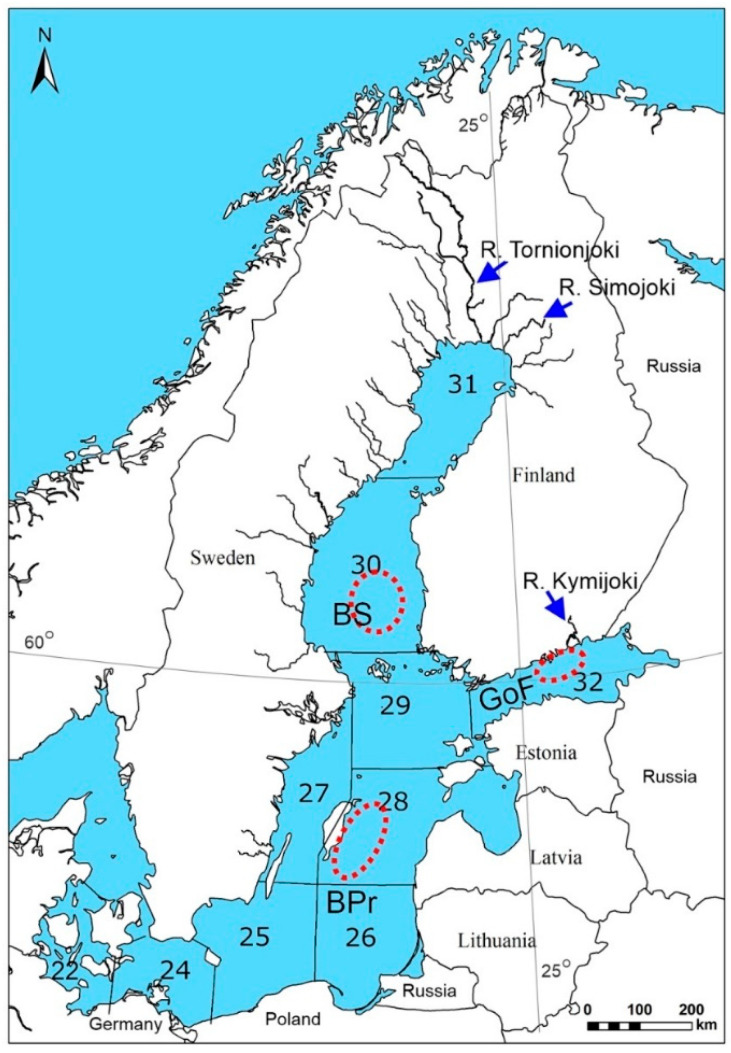
The three areas of the Baltic Sea where salmon (*Salmo salar*) were caught are depicted by broken-lined ellipses. The International Council for the Exploration of the Sea (ICES, origin of the map) subdivisions (SD) are presented: BPr = Baltic Proper (SDs 25–29), BS = Bothnian Sea (SD 30), and GoF = Gulf of Finland (SD 32). The Gulf of Bothnia consists of SDs 30 and 31. The Rivers Tornionjoki, Simojoki, and Kymijoki are indicated (blue arrows). The latitude of 60° and longitude of 25° are indicated.

The Atlantic salmon (*Salmo salar* L.) in the Baltic Sea (hereafter Baltic salmon or salmon) has been valuable in the human economy since prehistoric times. It has been easy to catch in rivers upon entry for spawning; open sea fishing started much later [1]. Organochlorine compounds have reduced the value of Baltic salmon as food, but the concentrations of polychlorinated biphenyls (PCBs) and dibenzo-p-dioxins (PCDD) have more than halved from those in the 1970s [2]. Due to the construction of power plant dams, water pollution, and the deterioration of spawning grounds by water level regulations and dredging, only 20 of the 80 salmon spawning rivers in the entire Baltic Sea remain. Most Baltic salmon come from the rivers of the Gulf of Bothnia, the most important of which is the River Tornionjoki (Figure 1). At the beginning of the 1990s, thiamine (vitamin B1) deficiency syndrome M74 erupted abruptly among Baltic salmon, meaning that most offspring of salmon that had been feeding in the Baltic Sea died on fish farms, as well as in Finnish and Swedish salmon rivers in the 1990s for several years, and a variable proportion of the fry since then [3,4,5,6]. M74 endangered the wild salmon stocks of the Baltic Sea, which had already weakened due to heavy open sea fishing in the 1980s. Although Baltic salmon stocks approached extinction in the 1990s [7], e.g., the salmon stock of the River Tornionjoki has recovered because of strict fishing restrictions and large-scale and expensive recovery measures [4,8]. However, many stocks need to be artificially maintained and strengthened, for which 4.5 million smolts are annually produced by fish farms. In recent years, the annual catch of salmon in the Baltic Sea region has been roughly 1000 tonnes [7].

Thiamine deficiency in salmonines, fatty fish, known as M74 in the Baltic Sea area and Thiamine Deficiency Complex (TDC) in North America, is associated with an abundant lipid-rich prey fish containing substantial amounts of polyunsaturated fatty acids (PUFAs) [9,10,11]. Thiamine deficiency particularly affects the offspring, which—during the embryonic and yolk-sac phases—must survive on the egg yolk nutrients provided by the brood female [3,12]. M74 and TDC symptoms, such as wriggling behavior and mortalities, have also been recorded among both sexes of ascended brood salmonines before and during the spawning period [4,13,14,15,16]. The reason for the sudden increase in M74 at the turn of the 1980s and 1990s was the over-fishing of cod (*Gadus morhua* L.), the principal predator of sprat (*Sprattus sprattus* (L.)). Consequently, the sprat stock exponentially increased and made an abundant food source of young fatty sprat available for salmon [8,17].

Triacylglycerols (TAGs) of lipids are the principal storage of metabolic energy in fish, and after TAG hydrolysis, the energy content of the liberated fatty acids (FAs) is converted via β-oxidation, the tricarboxylic acid cycle (TCA), the mitochondrial electron transfer chain, and oxidative phosphorylation into adenosine triphosphate (ATP) [18]. Thiamine is an essential micronutrient in energy metabolism [19]; its availability determines whether and how much ATP is produced [20,21]. Thiamine pyrophosphate (TPP) derivative functions as the cofactor of enzymes of the TCA: pyruvate dehydrogenase complex (PDHC), α-ketoglutarate dehydrogenase complex (KGDHC), and the branched chain α-keto acid dehydrogenase and of transketolase in the pentose phosphate shunt [22,23]. Thus, thiamine is also a key factor in sustaining the reducing power of cells, i.e., the nicotinamide adenine dinucleotide (NADH) and nicotinamide adenine dinucleotide phosphate (NADPH) status regulating FA and lipid metabolism [22,24]. TPP also functions as a coenzyme for 2-hydroxyacyl-CoA lyase (HACL1) in the α-oxidation of FAs [25].

Due to its central role in energy metabolism, the nutritional requirement of thiamine for humans, as well as for fish, is determined by dietary energy [21,26]. Because the net energy value of lipids is more than twice that of proteins, and, consequently, the boosted TCA consumes more thiamine [27], salmonines’ requirement of thiamine increases when they feed on lipid-rich fish [9]. The long-chain PUFAs of *n*−*3* family (*n*−*3* PUFAs), among which docosahexaenoic acid (DHA, 22:6*n*−*3*) has the highest number of double bonds, are extremely prone to lipid peroxidation [28,29]. Because lipid-rich marine fish as the diet increase both the body lipid content and the concentration of *n*−*3* PUFAs in the tissues of fatty fish species, the susceptibility of their tissues to lipid peroxidation increases [30,31,32]. The principal product of *n*−*3* PUFA peroxidation is malondialdehyde (MDA), which can be used as a biomarker of oxidative stress caused by lipid peroxidation [33,34,35,36]. As thiamine also has an antioxidant property that inhibits lipid peroxidation, its reserves are depleted in such antioxidant reactions [22,24,37].

The clupeid species sprat and herring (*Clupea harengus* L.) are the principal prey fish of Baltic salmon, but their prevalence differs between the basins of the Baltic Sea (Figure 1) [17,38,39]. The majority of salmon smolts from the rivers of the Gulf of Bothnia migrate to feed in the Baltic Proper (BPr) [40,41,42], where the proportion of sprat in the prey fish biomass of salmon has been larger than that of herring since the 1980s [17,43]. A smaller, annually variable proportion of salmon from the Bothnian Bay rivers halt to feed in the Bothnian Sea (BS) of the Gulf of Bothnia, where the dominant prey species is herring [17,43,44]. The proportion of salmon that remains to feed in the BS has depended on the smolt size and the strength of herring recruitment there; during 1986–2007, on average, approximately 14% of wild salmon smolts remained in the BS area annually instead of migrating to the BPr [45]. M74 also affects the salmon of the River Kymijoki, which flows into the Gulf of Finland (GoF, Figure 1) [4,8,46]. These salmon feed mainly in the GoF, where sprat has been more common than herring in recent decades [47,48]. However, prey fish, and, consequently, salmon, have been leaner in the GoF than in the BPr and BS, whereas they have been fattiest in the BS [49].

The average lipid content of sprat is almost twice that of herring of a similar size [9,50,51], i.e., the smallish herring that are of an appropriate size to be preyed on by salmon [17,39,52]. However, the proportion of PUFAs was or tended to be on average larger in herring than in sprat [10,50]. The concentration of the dominant PUFA, DHA, and, consequently, the total concentrations of *n*−*3* PUFAs and total PUFAs, increase with the increase in the lipid content of sprat and herring [10,50], as with marine fish in general [32]. Apart from the areas, the lipid content of sprat and herring varies with age, so that young sprat especially contain lipids in larger average percentages than older individuals [9]. The largest concentrations of DHA were therefore recorded in the youngest specimens in both species [10].

Thiamine concentrations of sprat and herring did not, in general, differ [9], but in the fall, when the lipid content of sprat and herring differed most, the thiamine concentration was smaller in sprat [51]. Since the thiamine concentration, in contrast to lipid content, was smallest in the youngest prey fish [9], the concentration of thiamine per unit of lipid and DHA was also on average smallest in them [9,10], but the differences in these concentrations in the different Baltic Sea areas have not been examined thus far. Like the lipid content, the proportion of TPP appeared larger in sprat than in herring caught in the fall [51], which suggests a positive relationship between the proportion of TPP and lipid content.

The concentrations and proportions of the thiamine components presumably change in relation to the dietary and body lipid content in salmon, as in sprat and herring. Their relationships could be investigated by analyzing the thiamine components and lipid content concurrently with the activity of glucose 6-phosphate dehydrogenase (G6PDH), which is a key regulatory enzyme in the lipogenic pathway and the principal NADPH-generating enzyme [53,54,55]. The activity of G6PDH is known to decrease with increasing dietary lipids in fish [53,54,55].

Thiamine deficiency itself increases oxidative stress [22,56,57], because TPP is needed in TCA cycle to sustain NADH and in the pentose phosphate shunt to generate NADPH. Thus, cellular defenses against oxidative stress are reduced [24]. Carotenoids, as powerful antioxidants, are apparently reduced when preventing general oxidative stress [58]. Fish, like other vertebrates, are unable to synthesize these pigments de novo, and they are mainly transferred to salmon via the stomach content of prey fish [59]. Thus, the concentration of carotenoids in salmon tissues during the feeding migration can indicate both the nutritional status of prey fish and the antioxidative capacity. Knowledge of carotenoid concentrations in feeding Baltic salmon and in prey fish of the different feeding areas of salmon is thus far lacking.

The concentrations of organochlorines vary in prey fish between the areas of the Baltic Sea, and the concentration differences are amplified in salmon [2,49,60,61]. In GoF salmon, the concentrations of PCBs and PCDDs in particular were larger than in salmon from the other Baltic Sea basins [2,49]. These two types of organochlorine are known to be among the most powerful inducers of hepatic 7-ethoxyresorufin-*O*-deethylase (EROD) activity in many fish species [34], and EROD activity has been widely used as a biomarker of exposure to these compounds, both in pollution monitoring and for environmental risk assessment [34,62,63,64]. Xenobiotics may also increase the content of reactive oxygen species (ROS) and thus oxidative stress in tissues [65], but MDA is principally generated in the peroxidation of *n*−*3* PUFAs [33,36,66]. We also analyzed the hepatic EROD activity to ensure that organochlorines, the concentrations of which in the studied salmon were known [49], had no association with thiamine status and the M74 syndrome.

Thiamine deficiency M74 in most cases develops as a result of feeding abundantly on sprat in the BPr [9,17], as was verified by the FA signature analysis (FASA) [3,10]. In 2004, only one out of 32 salmon female spawners from the River Simojoki (Figure 1) monitored for M74 was an evident M74 female, i.e., a female whose offspring suffered from M74. However, the FA composition of this female reflected the FAs of herring and FAs typical of the prey fish of the Gulf of Bothnia [10,67] compared with the FA composition of other females included in the FASA (Supplementary Figure S1) cf. [3]. The feeding area of this M74 female was also localized as being the BS, based on the ratio of PCDDs plus dibenzofurans (PCDD/Fs) to coplanar PCBs (CoPCBs) [2,52]. As the incidence of M74 is linked with the lipid and, therefore, also to the PUFA content of prey fish biomass [3,4,9,10], the abundant herring 2002 year class in the Gulf of Bothnia as prey [38] could have caused some M74 cases. Eating different foods could be detected by examining the dietary lipid-related biochemical indices of salmon caught in 2004 from the three different feeding grounds in the Baltic Sea.

The aim was to investigate, by means of differences in the nutritional quality of prey fish from the three sea areas, the association of the lipid and FA content of salmon diet with the biochemical parameters related to thiamine status. With this information, we were able to: (1) discover whether a diet rich in lipids and *n*−*3* PUFAs was already reflected in salmon thiamine status during their feeding phase in the sea; (2) elucidate the importance of peroxidation of *n*−*3* PUFAs in affecting salmon thiamine status; (3) clarify how the diet in the BS could cause such a severe thiamine deficiency that resulted in the M74 mortality of BS salmon offspring; and (4) confirm that organochlorines were not associated with the thiamine status of salmon. The hypothesis was that the differences in the lipid and PUFA content of prey fish between the three feeding grounds manifested themselves in the muscular and hepatic biochemical indices of salmon that were physiologically related to lipid content, lipid peroxidation, and thiamine status. This would demonstrate that the peroxidation of *n*−*3* PUFAs in general affects thiamine status in fatty predatory fish, and that a large amount of prey fish lipids with long-chain *n*−*3* PUFAs rather than a specific prey fish species is to blame for salmonine thiamine deficiency.

## 2. Materials and Methods

### 2.1. Fish and Sample Preparation

#### 2.1.1. Prey Fish

Sprat (*Sprattus sprattus* (L.)) and herring (*Clupea harengus* L.) were collected from commercial midwater trawl catches from the northern BPr, the BS, and the GoF during the late fall of 2003 (fall) and late spring of 2004 (spring), as described in Keinänen et al. [10]. Because fish are fattiest in the late fall and leanest in the late spring [9], prey fish combined in this way were believed to represent the average diet. Sprat were also sampled from the BS despite its marginal role in this area as prey for salmon [17,43,44]. Immediately after capture, the total length and weight of each fish specimen were measured, the otoliths were removed for age determination [68], and the fish were individually sealed in numbered ziplock bags and frozen at −20 °C. Since adult salmon prefer prey fish of 4–15 cm in total length and only rarely prey on fish > 20 cm [39,52], all age groups of sprat and the age groups of herring with a total length < 19 cm are considered suitable prey for salmon [17]. Only such herring specimens were, therefore, included in this study. In the laboratory, age groups 1, 2–3, and 6–8 of sprat (in total 544 specimens) and 1, 2, 3, and 6 of herring (in total 1280 specimens) were selected for pooling, except for 1-year-old sprat from the BS in the spring, which were missing, probably because sprat do not spawn in the area, and the youngest sprat had not yet moved there from the BPr [41]. All fish specimens belonging to the same species, area, age group, and season were pooled (7–133 specimens per pool) for homogenization (Supplementary Table S1) as whole fish to simulate the salmon diet, as in previous studies [9,10,49,51], and kept deep-frozen (−80 °C, ULT Freezer Forma 905, Thermo Electron Corp., Marietta, OH, USA). Subsamples were taken from each homogenized whole-body pool of sprat (17 pools) and herring (24 pools) for the analysis of the concentrations of the thiamine components and carotenoids, whereas the total lipid content of the pools (hereafter lipid content) and the FA concentrations were provided by Keinänen et al. [10].

#### 2.1.2. Salmon

Salmon (*Salmo salar* L.) were caught with drift nets by commercial fishermen during the feeding migration in the late fall of 2004 from the BPr, the BS, and the GoF (Figure 1). The caught immature salmon (BPr 9, BS 5, and GoF 10 specimens) were in their first to third feeding seasons at sea. A 10 g sample of the posterior liver of a stunned salmon, except for five salmon from the GoF, was dissected and immediately frozen between slices of dry ice. Liver samples were transported in dry ice to the laboratory and preserved in liquid nitrogen until they were analyzed. The whole fish were individually sealed in polyethylene bags and frozen (−20 °C for 2 weeks). In the laboratory, the total length and body weight of each salmon (in total 24 specimens) were measured (Table 1), and scales were removed from above the lateral line below the dorsal fin to determine age [69]. A muscle sample (approximately 40 g) was taken from epaxial white muscle from below the dorsal fin of the frozen whole fish and immediately refrozen at −80 °C for the analyses. The rest of the salmon was homogenized to analyze the halogenated organic compounds and total body lipids [49]. Both males and females were included in the study, because the sex of non-spawned immature salmon was not expected to affect the results for the analyzed parameters. In the salmon liver (in total 19), the concentrations of the thiamine components, the concentration of malondialdehyde (MDA) [36], and the activities of glucose 6-phosphate dehydrogenase (G6PDH) and 7-ethoxyresorufin-*O*-deethylase (EROD) were analyzed. In the salmon muscle (samples, in total 24, kept deep-frozen at −80 °C for 4–6 weeks), the concentrations of thiamine components, total carotenoids, and FAs were analyzed. The whole-body total lipid content (hereafter lipid content) and organochlorine concentrations were obtained from Vuorinen et al. [49].

### 2.2. Chemical Analyses

#### 2.2.1. Analysis of Thiamine Components

Thiamine components were analyzed in the liver and muscle of salmon and pools of sprat and herring, each species separately, by high-performance liquid chromatography (HPLC), as described in Vuorinen et al. [4]. Thiamine components comprised the two phosphorylated thiamine derivatives, thiamine monophosphate (TMP) and thiamine pyrophosphate (TPP), as well as unphosphorylated thiamine (THIAM), which were summed up as total thiamine (TotTh).

#### 2.2.2. Analysis of Carotenoids

The total carotenoid concentration was determined in the salmon muscle and the pools of sprat and herring, each species separately. The concentrations were measured spectrophotometrically according to Pettersson and Lignell [70], with slight modifications, described in Vuorinen et al. [71].

#### 2.2.3. Fatty Acid Analyses

FAs were analyzed as described in Keinänen et al. [10]. The FA results for salmon are presented as lipid-weighted proportions, which correspond to the concentrations (mg g^−1^). The FAs were grouped into their structural categories (later FA classes): saturated FAs (SFAs), monounsaturated FAs (MUFAs), and PUFAs, and the latter were further divided into *n*−*3* PUFAs and *n*−*6* PUFAs, similarly to Keinänen et al. [3,10]. DHA constituted on average 47% of PUFAs and 58% of *n*−*3* PUFAs in salmon. The 16 FAs (Supplementary Table S2), for which the mean proportion of the total chromatogram peak area was >0.4%, were included in the calculations.

#### 2.2.4. Enzyme Activity and Malondialdehyde Determinations

Hepatic microsomes were prepared to determine EROD activity, and supernatant was used to measure the MDA concentration as the indicator of lipid peroxidation and to determine the activity of G6PHD, as described in Vuorinen et al. [71].

#### 2.2.5. Quality Control

The quality of the analyses was assured through analysis of parallel samples and using the laboratory control samples.

### 2.3. Calculations and Statistical Analyses

The Pearson correlation analysis was applied to test the relationships of the lipid content and the concentrations of FA classes in salmon, with the concentration and proportion of the muscular and hepatic thiamine components and hepatic MDA, and the activities of G6PDH and EROD. Pearson correlations were also calculated between lipid content and thiamine component concentrations, and proportions of sprat and herring. Variables were tested for normality (Kolmogorov–Smirnov test), and Levene’s test was used to test homogeneity among variances. One-way ANOVA with the post hoc test of Student Newman–Keuls (SNK) was used to reveal significant (*p* < 0.05) spatial differences in the biochemical proxies of liver and muscle of salmon, and the percentage of lipid content. Differences in the concentrations and proportions of TotTh and its components between sprat and herring in the fall and spring were similarly tested. Testing for salmon parameters was also performed pairwise, i.e., the differences between biochemical proxies of each pair of the sampling locations were tested applying a t-test. Linear regression was applied in analyzing the relationships of the lipid, *n*−*3* PUFA, and DHA content of salmon with the lipid content of prey fish sampled in the fall. Linear regressions were also fitted for the relationships between the biochemical proxies of liver and muscle of salmon and the percentage of lipid content, and for the relationships between the age of prey fish and lipid content and carotenoid, TotTh, and FA structural class concentrations in the three sea areas. MANOVA followed by LsMeans at a significance level of *p* < 0.05 was applied to analyze the species (sprat and herring), and the spatial (the northern BPr, the BS, and the GoF) and seasonal (fall and spring) dependencies of biometric and biochemical factors of prey fish.

A principal component analysis (PCA) was carried out [72] for multivariate statistical comparisons of biochemical parameters in salmon. As a result, a biplot graph with principal components PC1 and PC2 was created to demonstrate (dis)similarities among the salmon and to indicate correlations between the variables. Soft independent modeling of class analogy (SIMCA) was used to quantify the differences at the level *p* < 0.05 between the pairs of salmon groups [73].

The statistical analyses were performed with the Statistical Analysis System software (ver. 9.4, SAS Institute Inc., Cary, NC, USA) [74], apart from PCA and SIMCA, which were carried out using Sirius software [ver. 8.5, Pattern Recognition Systems (PRS), Bergen, Norway]. Figures were drawn with Origin Pro 2021 (OriginLab Co., Northampton, MA, USA).

## 3. Results

### 3.1. Nutritional State

#### 3.1.1. Nutritional Characteristics of Prey Fish

**Figure 2:**
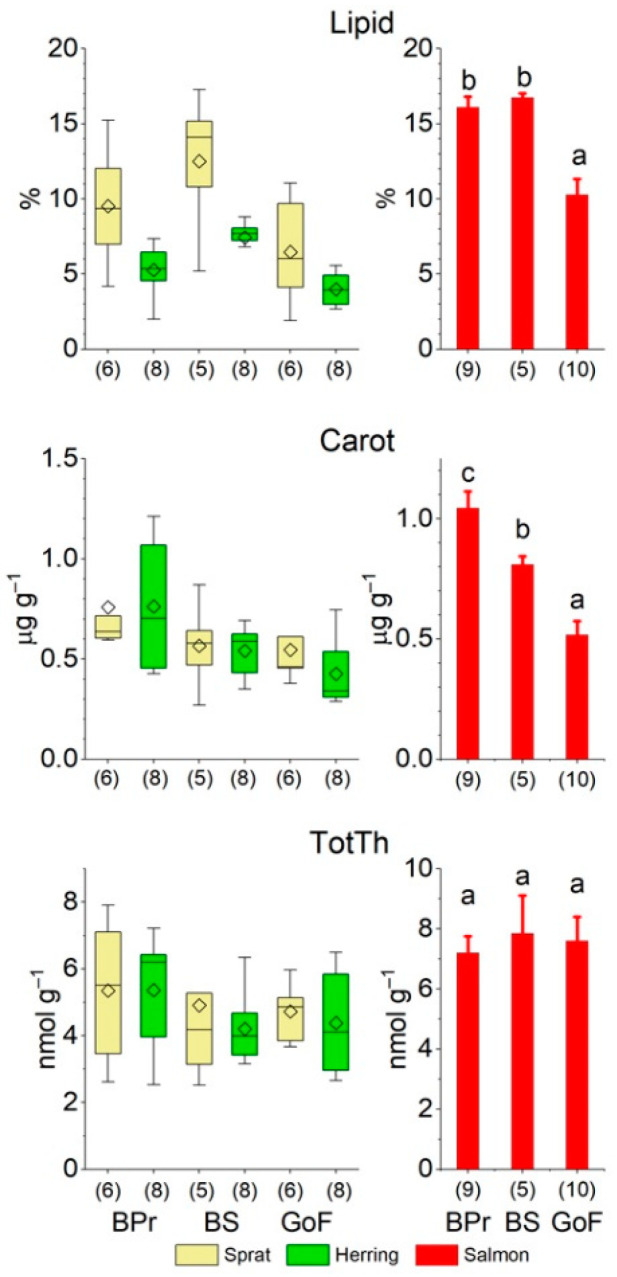
Total lipid content (Lipid) in the whole body of sprat (*Sprattus sprattus*) and herring (*Clupea harengus*) as box plots (with the samples of different ages in the fall and spring combined) and salmon (*Salmo salar*) (right column, mean ± SE, in the late fall), and the concentrations of total carotenoids (Carot) and total thiamine (TotTh) in sprat and herring, and in the muscle of salmon, from the three areas of the Baltic Sea, the Baltic Proper (BPr), the Bothnian Sea (BS), and the Gulf of Finland (GoF). Different letters denote significant (*p* < 0.05) differences between the areas in salmon. In the box plots, whiskers depict 1 × SD, upper and lower parts of the boxes 25 and 75% of observations, horizontal lines the median value, and diamonds the mean concentrations. The number of observations is given in parentheses; sprat and herring pools are each composed of 7–133 individual fish.

Lipid content was significantly greater in sprat than in herring, and, in general, in prey fish largest in the BS and smallest in the GoF (Figure 2, Supplementary Tables S3 and S4). Prey fish in the GoF were significantly leaner than in the other areas according to MANOVA (Supplementary Table S4). In addition, the lipid content of prey fish was significantly larger in the fall than in the spring (Supplementary Table S4 and Figure S2). In sprat, lipid content decreased significantly with age, especially in the fall, while the age relationship in herring was unclear—the lipid content of herring in the BPr increased with age in the fall and decreased in the spring (Supplementary Figure S2).

The total carotenoid concentration was similar in both prey fish species but differed between the areas (Figure 2, Supplementary Tables S3 and S4); it was significantly larger in the BPr than in the GoF and BS. There was no age dependence in carotenoid concentrations in prey fish (Supplementary Figure S2).

The TotTh concentrations of prey fish did not differ between species, areas, or seasons (Figure 2, Supplementary Tables S3 and S4). In most cases, the TotTh concentration increased or tended to increase with age in prey fish (Supplementary Figure S2).

**Figure 3:**
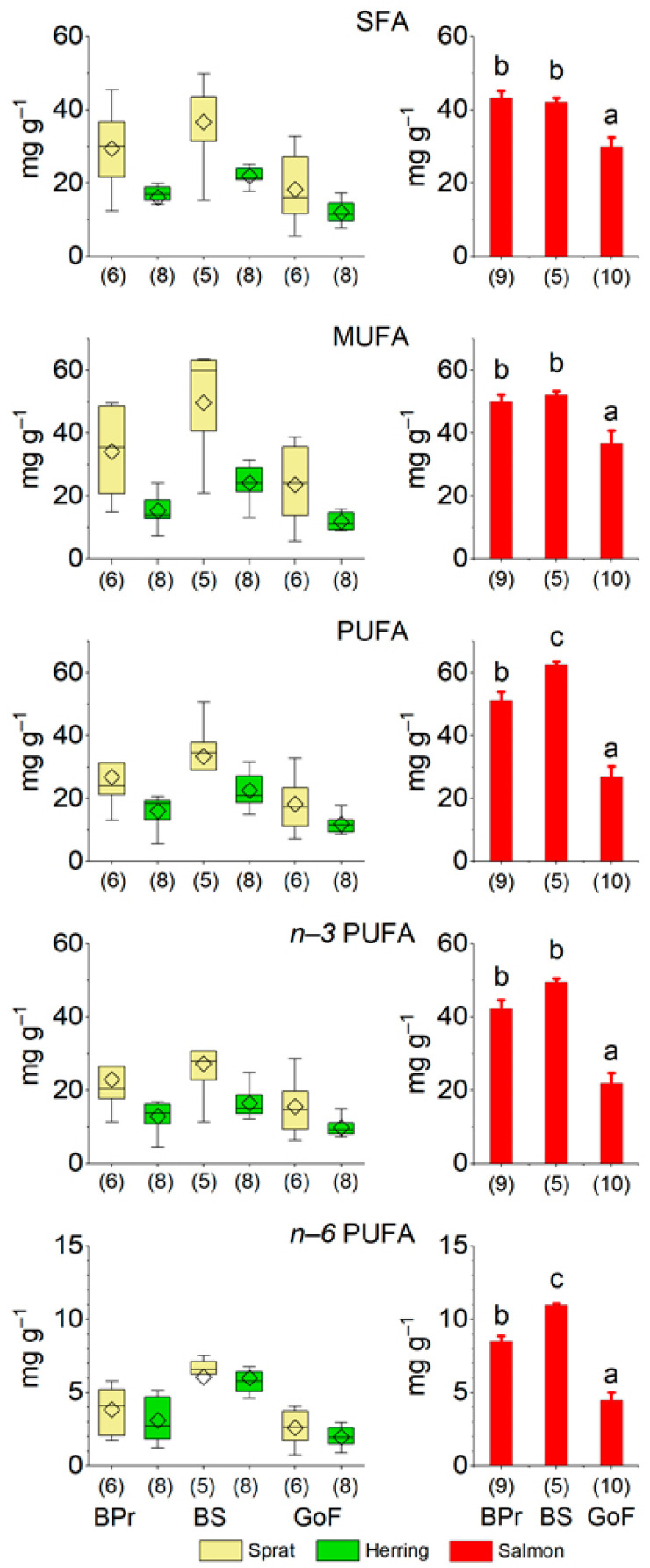
Concentrations of SFAs, MUFAs, PUFAs, and *n*−*3* and *n*−*6* PUFAs in the whole body of sprat (*Sprattus sprattus*) and herring (*Clupea harengus*) (with the samples of different ages in the fall and spring combined) as box plots, and in salmon (*Salmo salar*) (right column, mean ± SE, in the late fall), from the three areas of the Baltic Sea, the Baltic Proper (BPr), the Bothnian Sea (BS), and the Gulf of Finland (GoF). For further details, see the legend for Figure 2.

The concentrations of all FA classes, except for *n*−*6* PUFAs, were significantly larger in sprat than in herring (Figure 3, Supplementary Tables S3 and S4). Considering the areas, the concentrations of SFAs, *n*−*6* PUFAs, and total PUFAs in the prey fish in general differed significantly between all study areas and were largest in the BS and smallest in the GoF. The MUFA concentration was, likewise, largest in the prey fish in the BS but similar in the BPr and GoF, and the concentration of *n*−*3* PUFAs was significantly smaller in the prey fish in the GoF than in the other areas (Supplementary Tables S3 and S4). Moreover, the concentrations of all FA classes were larger in the fall than in the spring (Supplementary Table S4). In general, the concentrations of SFAs, MUFAs, and PUFAs and their structural families in sprat decreased with age in both the fall and spring (Supplementary Figure S3). In herring, the age relationship of the concentrations of FA classes varied depending on the sea area and season; for PUFAs and the structural families they contained the tendency was to decrease, and for MUFAs to increase. Especially in BS herring, the concentration of *n*−*3* PUFAs (Supplementary Figure S3) and the DHA concentration (not shown) were, therefore, largest in the youngest individuals.

#### 3.1.2. Reflection of Prey Fish Nutrients in Salmon

Consistent with that found for the prey fish, salmon had the smallest lipid content in the GoF, but the difference in the lipid content between BPr salmon and BS salmon was not significant (Figure 2). The total carotenoid concentration in salmon muscle was, as in prey fish, largest in the BPr and smallest in the GoF, and the difference was significant between all three areas (Figure 2). Moreover, as with prey fish, the TotTh concentrations in the muscle of salmon did not differ between the three study areas (Figure 2).

The concentrations of all FA classes in salmon, as in prey fish, were significantly smaller in the GoF than in the BPr and BS (Figure 3). In addition, salmon from the BPr and BS differed significantly in relation to the concentration of total PUFAs and *n*−*6* PUFAs, which were larger in BS salmon (Table 1), similarly to prey fish. In pairwise testing, the concentration of *n*−*3* PUFAs in salmon was also significantly (*p* < 0.05) larger in the BS than in the BPr. The DHA concentration also tended to be larger in BS salmon than in BPr salmon (Table 1), but the difference was not significant. In GoF salmon, the DHA concentration was significantly smaller than in the salmon of the other two areas (Table 1), whereas in prey fish the difference was only significant between the GoF and BS.

**Table 1:** Mean (±SE) concentrations (i.e., lipid-weighed proportions) of fatty acids (FAs, mg g^−1^), their structural classes, and some of their ratios in salmon (*Salmo salar*) from the Baltic Proper (BPr, *N* = 9), Bothnian Sea (BS, *N* = 5), and Gulf of Finland (GoF, *N* = 10). The mean weight and length with the ranges are also given.

	Baltic Proper (BPr)			Bothnian Sea (BS)			Gulf of Finland (GoF)			*p*	*F*_2,21_
Saturated FAs (SFA)											
14:0	5.82	±	0.28 ^b^	5.31	±	0.24 ^b^	2.88	±	0.31 ^a^	<0.0001	30.6
16:0	29.67	±	1.32 ^b^	29.49	±	0.84 ^b^	20.10	±	1.78 ^a^	0.0002	13.1
17:0	0.81	±	0.04 ^b^	0.87	±	0.02 ^b^	0.54	±	0.03 ^a^	<0.0001	25.6
18:0	5.56	±	0.26	5.66	±	0.15	5.49	±	0.42	0.950	0.05
SFA	43.20	±	1.93 ^b^	42.11	±	1.20 ^b^	29.92	±	2.56 ^a^	0.0004	11.5
Monounsaturated FAs (MUFA)											
16:1*n*−*7*	6.91	±	0.27 ^b^	7.38	±	0.18 ^b^	5.08	±	0.64 ^a^	0.010	5.86
17:1*n*−*8*	1.00	±	0.04 ^b^	0.69	±	0.04 ^a^	0.62	±	0.05 ^a^	<0.0001	22.1
18:1*n*−*9*	34.85	±	1.59 ^b^	32.97	±	1.11 ^b^	25.01	±	2.94 ^a^	0.013	5.34
18:1*n*−*7*	4.84	±	0.22 ^a^	7.74	±	0.34 ^b^	4.15	±	0.36 ^a^	<0.0001	26.8
20:1*n*−*9*	1.74	±	0.26	2.24	±	0.10	1.39	±	0.25	0.116	2.39
MUFA	50.03	±	2.22 ^b^	52.14	±	1.16 ^b^	36.71	±	4.11 ^a^	0.007	6.45
Polyunsaturated FAs (PUFA)											
18:2*n*−*6*	5.33	±	0.26 ^b^	7.58	±	0.13 ^c^	2.29	±	0.34 ^a^	<0.0001	68.6
18:3*n*−*3*	4.25	±	0.21 ^b^	4.29	±	0.14 ^b^	1.66	±	0.24 ^a^	<0.0001	47.5
20:2*n*−*6*	1.19	±	0.07 ^b^	2.14	±	0.04 ^c^	0.61	±	0.08 ^a^	<0.0001	86.0
20:4*n*−*6* (ARA)	0.96	±	0.04	1.24	±	0.03	0.93	±	0.12	0.113	2.42
20:5*n*−*3* (EPA)	9.36	±	0.54 ^b^	9.91	±	0.30 ^b^	4.20	±	0.57 ^a^	<0.0001	33.8
22:5*n*−*3* (DPA)	4.58	±	0.23 ^b^	5.51	±	0.20 ^b^	2.99	±	0.36 ^a^	<0.0001	15.7
22:6*n*−*3* (DHA)	23.82	±	1.49 ^b^	28.53	±	0.71 ^b^	12.91	±	1.68 ^a^	<0.0001	24.6
PUFA	51.20	±	2.73 ^b^	62.53	±	1.13 ^c^	26.86	±	3.44 ^a^	<0.0001	33.0
*n*−*3* PUFA	42.25	±	2.37 ^b^	49.53	±	0.96 ^b^	21.94	±	2.85 ^a^	<0.0001	29.4
*n*−*6* PUFA	8.47	±	0.40 ^b^	10.96	±	0.12^c^	4.47	±	0.57 ^a^	<0.0001	41.1
Weight, kg	3.6 (2.6–4.6)			3.7 (3.1–4.5)			2.7 (1.1–6.2)				
Length, cm	71.1 (64.3–77.8)			70.9 (67.4–74.3)			60.1 (49.3–85.8)				

The *p*-value and the *F*-value in one-way ANOVA are given; a different letter as a superscript to SE indicates a significant difference (*p* < 0.05, Student Neuman–Kuels post hoc test) between the means of the three sea areas.

#### 3.1.3. Thiamine per Lipid and PUFA in Prey Fish and Salmon

**Figure 4:**
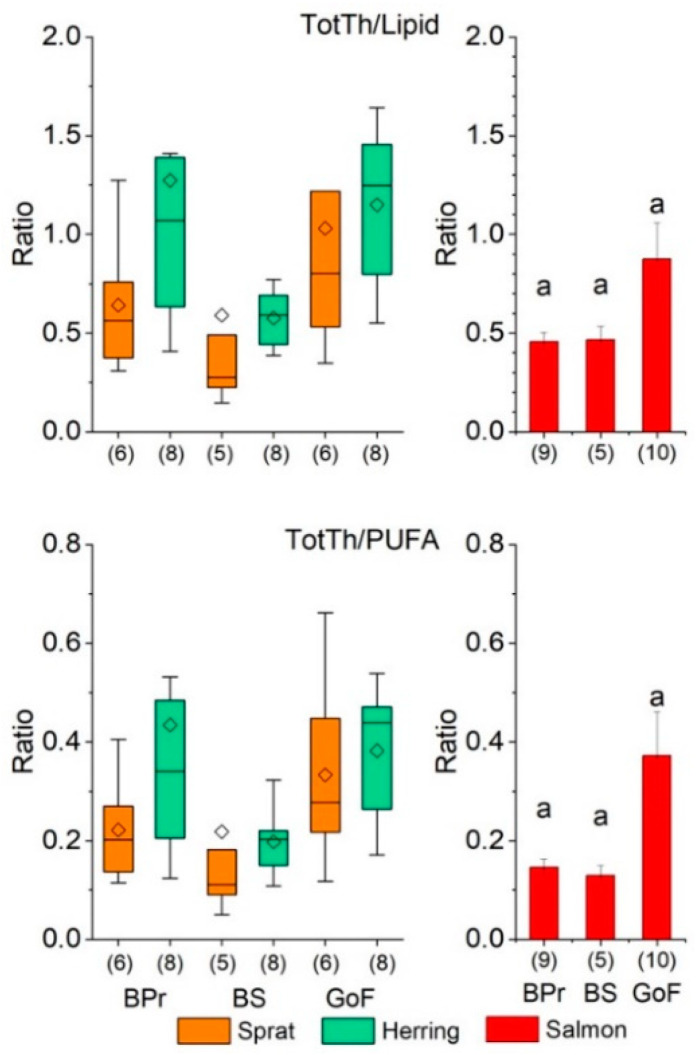
Ratios of total thiamine concentration to total lipid (TotTh/Lipid) and PUFA (TotTh/PUFA) concentration in the whole body of sprat (*Sprattus sprattus*) and herring (*Clupea harengus*) (with the samples of different ages in the fall and spring combined), and salmon (*Salmo salar*) (TotTh in muscle and lipids in the whole body) from the Baltic Proper (BPr), the Bothnian Sea (BS), and the Gulf of Finland (GoF). For further details, see the legend for Figure 2.

The ratio TotTh/lipid content was significantly smaller in prey fish in the BS compared to the GoF and tended to be smaller in the BS than in the BPr, although the difference was not significant between the BPr and GoF or the BPr and BS (Figure 4, Supplementary Tables S3 and S4). In the BPr, the ratio TotTh/lipid content was twice as high in herring as in sprat in the combined fall and spring data, whereas in the BS and GoF, the mean of ratios did not differ between the prey species (Supplementary Table S3). The ratio TotTh/lipid content in general increased significantly or tended to increase as a function of age in both prey species, although not in herring in the BS and GoF in the spring (Supplementary Figure S4).

Prey fish tended to have the smallest TotTh/PUFA ratio in the BS and the largest in the GoF, and the ratio in general in prey fish differed significantly between the seasons and was larger in the spring (Figure 4 and Supplementary Table S4). The ratio TotTh/PUFA increased as a function of age in both prey species in all areas—except for BPr sprat in the fall and both species in the GoF in the spring. Although the relationship was not significant in most of the cases (Supplementary Figure S4), the large values for the coefficient of determination indicated the models’ good fit.

Although the differences in the mean TotTh/lipid content and TotTh/PUFA ratio in salmon between the areas were not significant, the ratios tended, similarly to sprat and herring, to be smallest in the BS and largest in the GoF (Figure 4).

### 3.2. Thiamine Components

#### 3.2.1. Thiamine Components in Prey Fish

In prey fish, THIAM comprised the largest thiamine component, and TMP the smallest (Supplementary Table S3 and Figure S5). The concentrations of TPP and TMP in general were significantly smaller in prey fish in the GoF than in the BPr. The concentrations of both phosphorylated thiamine derivatives were significantly larger in the fall than the spring, and the concentration of TMP was significantly larger in sprat than in herring (Supplementary Table S4).

#### 3.2.2. Thiamine Components in Salmon

The concentration of TotTh was several times larger in the liver than in the muscle of salmon, and in the liver, it differed significantly between all areas (Table 2). TPP comprised the largest component of thiamine in both tissues in salmon of all areas, whereas THIAM comprised the smallest part in both tissues (Table 2, Supplementary Figure S6).

The proportion of TPP in salmon muscle differed significantly between the three study areas: It was largest in the BS (84%) and smallest in the GoF (51%) (Supplementary Figure S6). In contrast, the proportion of muscle THIAM was smallest in the BS (1%) and largest in the GoF (12%). The differences in the concentrations of TPP and THIAM in muscle were parallel to their proportions (Supplementary Figure S6). The concentration (Table 2) and proportion (Supplementary Figure S6) of TMP in muscle were smaller than those of TPP, although in the leanest fish, in the GoF, the differences between TMP and TTP were not large.

In the liver, both the TotTh and TPP concentration were largest in the GoF and smallest in the BS (Table 2, Supplementary Figure S6). The concentration of hepatic TMP was smallest in the BS, where the concentration of hepatic THIAM was largest among the areas, although it was very small overall (Table 2). In the BS, the proportion of hepatic THIAM was 3.3%, whereas in the GoF, it was 0.4% (Supplementary Figure S6).

### 3.3. Salmon Liver Biochemistry

**Figure 5:**
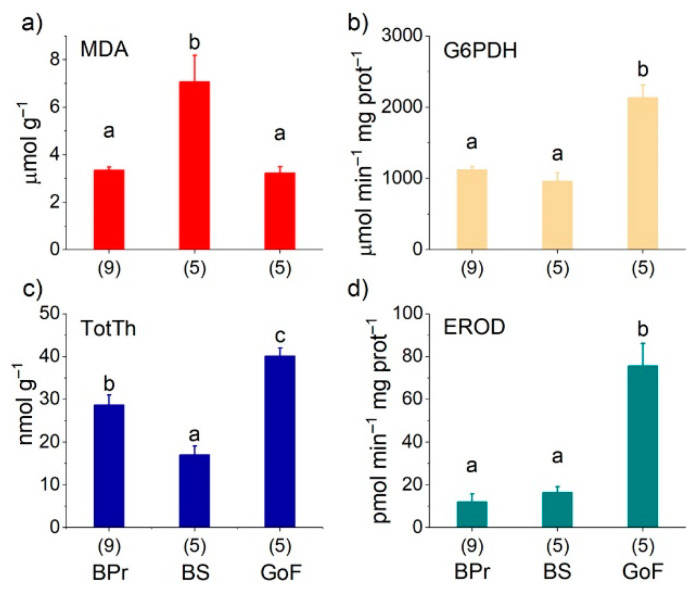
Mean (±SE) (**a**) malondialdehyde (MDA) concentration, (**b**) glucose 6-phosphate dehydrogenase (G6PDH) activity, (**c**) total thiamine (TotTh) concentration, and (**d**) 7-ethoxyresorufin-*O*-deethylase (EROD) activity in the liver of salmon (*Salmo salar*) in the Baltic Proper (BPr), the Bothnian Sea (BS), and the Gulf of Finland (GoF) in the late fall. Different letters denote statistically significant (*p* < 0.05) differences between the areas. The number of salmon is given in parentheses.

The hepatic MDA concentration was significantly greater in BS salmon than in GoF and BPr salmon (Figure 5). In contrast, the TotTh concentration of the liver was smallest in BS salmon but differed significantly between all the areas, being largest in salmon of the GoF. Likewise, the hepatic G6PDH activity was significantly higher in GoF salmon than in salmon from the other two areas (Figure 5).

### 3.4. Relationships between the Indices

#### 3.4.1. Relationship of Salmon Lipid and Fatty Acid Content with Prey Fish Lipid Content

**Figure 6:**
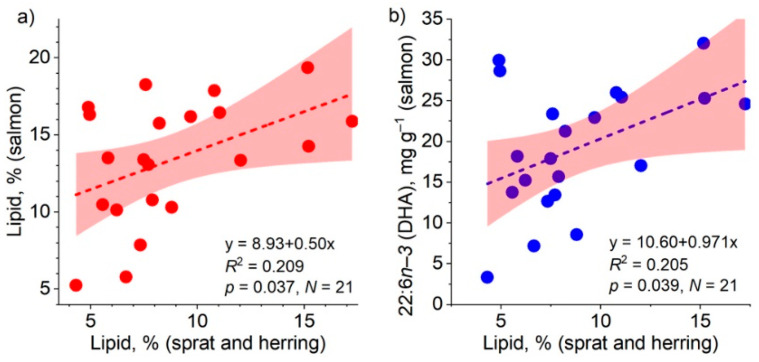
Relationship of the (**a**) lipid content and (**b**) docosahexaenoic acid (22:6*n*−*3*, DHA) concentration of salmon (*Salmo salar*) with lipid content of prey fish sprat (*Sprattus sprattus*) and herring (*Clupea harengus*) caught in the fall. Salmon, sprat, and herring were from the Baltic Proper, Bothnian Sea, and Gulf of Finland. The fitting of the linear models with 95% confidence bands, coefficients of determination (*R*^2^), significances (*p*) of the model, and the number of samples are given.

The lipid content, and likewise the concentration of DHA, in salmon increased significantly with the increase in the lipid content of prey fish when all salmon and prey fish (sprat and herring) caught in the fall were included in the analysis (Figure 6). The respective relationship was also significant for *n*−*3* PUFAs (*R*^2^ = 0.201, *p* = 0.041, *N* = 21).

#### 3.4.2. Relationship of Thiamine and Its Components with Lipid Content and Fatty Acid Content within Species

**Figure 7:**
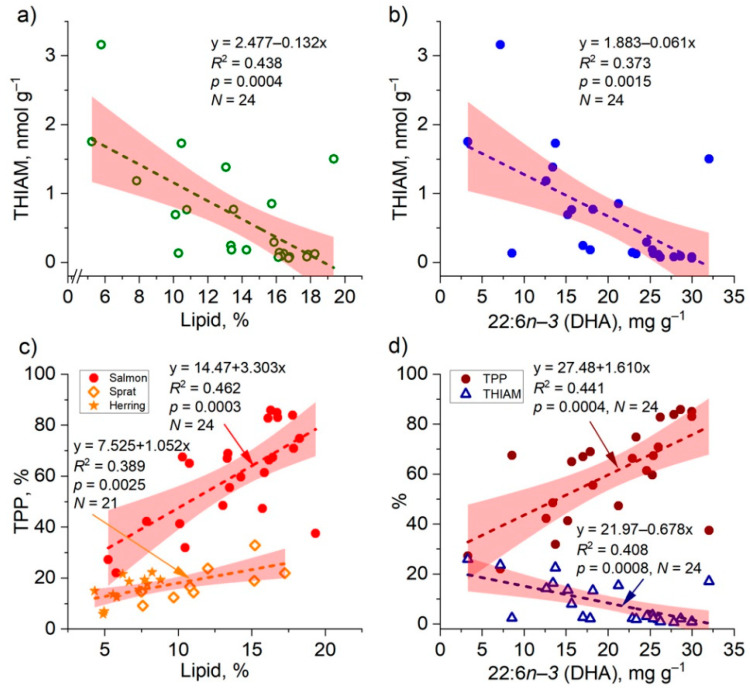
The relationship of the concentration of muscle free thiamine (THIAM) of salmon (*Salmo salar*) with (**a**) total lipid content and (**b**) docosahexaenoic acid (22:6*n*−*3*, DHA) concentration, (**c**) relationship of the proportion of thiamine pyrophosphate (TPP) in salmon muscle and in the whole body of sprat (*Sprattus sprattus*) and herring (*Clupea harengus*) with total body lipid content, and (**d**) relationship of the proportions of muscle TPP and THIAM with the DHA concentration of salmon. Salmon, sprat, and herring were from the Baltic Proper, Bothnian Sea, and Gulf of Finland. The fitting of the linear models with 95% confidence bands, coefficients of determination (*R*^2^), significances (*p*) of the model, and the number of samples are given.

The concentration and proportion of muscle THIAM in salmon was correlated highly significantly and negatively with the lipid content, as was the concentration of TMP (Table 3). An increase of 1% in lipid content would thus result in a decrease of 0.13 nmol g^−1^ in muscle THIAM according to the linear model (Figure 7). In sprat, the concentrations of THIAM and lipid were likewise significantly and negatively correlated in the total dataset (Table 3) and in the fall (r = −0.806, *p*< 0.01, *N* = 9). In both prey species, the proportion of THIAM were significantly and negatively correlated with their lipid content (Table 3).

In contrast to THIAM, the concentration and proportion of muscle TPP in salmon was positively correlated with the lipid content (Table 3). In herring, the concentrations of TPP and TMP were positively correlated with lipid content, and tended to be correlated in sprat, and the correlation of TPP proportion with lipid content was significant in both prey species (Table 3). In salmon muscle, and in prey fish in the fall, the proportion of TPP increased according to a linear model as a function of the lipid content (Figure 7).

The proportion of TPP in salmon muscle increased with increasing PUFA, *n*−*3* PUFA, and DHA concentration, while that of THIAM decreased (Table 3 and Figure 7).

#### 3.4.3. Relationship of Liver Biochemical Indices with Lipid and Fatty Acid Content in Salmon

**Figure 8:**
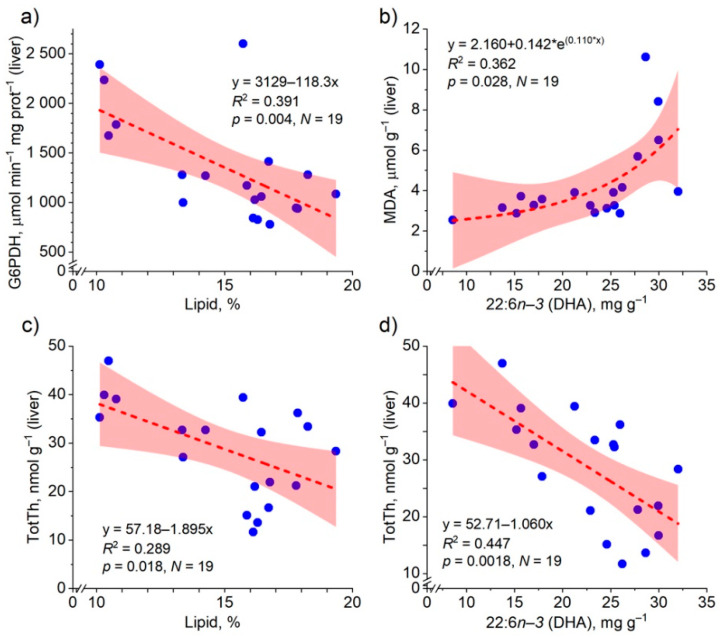
Relationships of the (**a**) hepatic glucose 6-phosphate dehydrogenase (G6PDH) activity with whole-body lipid content, (**b**) hepatic malondialdehyde (MDA) with docosahexaenoic acid (22:6*n*−*3*, DHA) concentration, (**c**) hepatic total thiamine (TotTh) concentration with whole-body lipid content, and (**d**) hepatic TotTh concentration with DHA concentration of salmon (*Salmo salar*) from the Baltic Proper, Bothnian Sea, and Gulf of Finland. The fitting of the linear models with 95% confidence bands, coefficients of determination (*R*^2^), significances (*p*) of the model, and the number of samples are given.

The hepatic G6PDH activity was strongly and negatively correlated with the lipid content (Table 4), and the decrease was linear with the increase in lipid content (Figure 8). The G6PDH activity was also correlated or tended to be correlated negatively with the concentrations of all FA classes (Table 4). Conversely, the MDA concentration had a significant and positive correlation only with the concentrations of hepatic PUFAs and the structural families they contained, and it was not correlated with the lipid content and the concentrations of SFAs or MUFAs (Table 4). The increase in the MDA concentration with the increase in the DHA concentration was exponential (Figure 8), as it was with the PUFA concentration (not shown).

The TotTh concentration of salmon liver had a significant negative correlation with the lipid content, and it was also strongly and negatively correlated with the concentration of PUFAs and their structural families, but not at all with SFAs and not significantly with MUFAs (Table 4). The decrease in the hepatic TotTh concentration with the increase in the lipid content and the DHA concentration was linear (Figure 8), and according to the model, a 1 mg g^−1^ increase in the DHA concentration caused on average 3.6% decrease in the hepatic TotTh concentration, while with the respective increase in the PUFA concentration, the TotTh concentration decreased by 1.8%. The hepatic TotTh concentration was not correlated with the concentration of the major MUFA, 18:1*n*−*9* (*r =* −0.344, *p =* 0.150, *N* = 19).

#### 3.4.4. Carotenoid Relationships in Salmon

The carotenoid concentration of salmon muscle was positively correlated with lipid content (*r* = 0.553, *p* = 0.005) and the PUFA and *n*−*3* PUFA concentrations (*r* = 0.413, *p* = 0.045 and *r* = 0.428, *p* = 0.037, respectively), but it was not correlated with the thiamine concentrations or proportions. Nor was it correlated with the hepatic MDA concentration (*r* = −0.079, *p* = 0.748).

#### 3.4.5. Organochlorine Relationships with the Hepatic Parameters in Salmon

EROD activity was significantly and several times larger in GoF salmon than in salmon from the two other areas. The latter two did not differ significantly (Figure 5). There were significant positive correlations between EROD activity and the concentrations (in fresh weight) of PCBs (*r* = 0.631, *p* = 0.004) and PCDDs (*r* = 0.488, *p* = 0.034), but not with PCDFs and CoPCBs, or toxic equivalents in total or those of PCDDs, PCDFs, or PCBs.

The hepatic MDA and TotTh concentrations were not correlated with any of the organochlorine sum concentrations. The G6PDH activity was positively and significantly correlated with the total PCB concentration (*r* = 0.485, *p* = 0.035). The data are not shown.

### 3.5. Spatial Differences in Salmon Biochemistry

**Figure 9:**
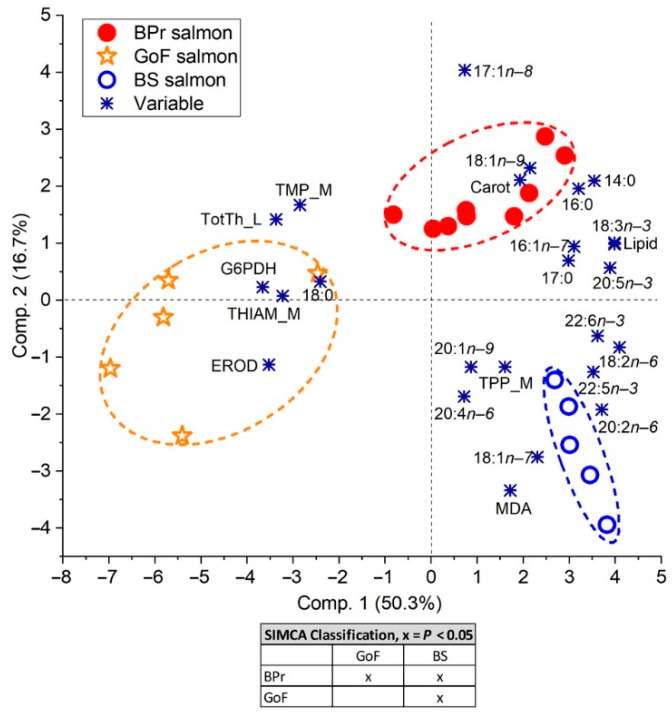
Biplot from the PCA model with principal components 1 and 2 for salmon (*Salmo salar*) caught in the Baltic Proper (BPr), the Bothnian Sea (BS), and the Gulf of Finland (GoF) in the late fall, and the variables measured: the activity of glucose 6-phosphate dehydrogenase (G6PDH) and 7-ethoxyresorufin-*O*-deethylase (EROD) and the concentration of malondialdehyde (MDA) and total thiamine in liver (TotTh_L), the concentrations of thiamine components (TPP = thiamine pyrophosphate, TMP = thiamine monophosphate and THIAM = unphosphorylated or free thiamine) in the muscle (M), the concentration of total carotenoids (Carot) and the concentrations of individual fatty acids, and the lipid content (Lipid) in the whole body. Results of the paired SIMCA test for salmon from the three Baltic Sea areas are also presented.

Salmon in the three study areas differed significantly based on all measured biochemical characteristics according to the PCA and SIMCA analyses (Figure 9). In the PCA model, PC1 seemed to represent the body content of lipids, the FAs they contained, and lipophilic substances, and explained 50% of total data variation, and PC2 additionally separated samples based on pelagic versus benthic influences on the diet (explaining 17% of the variation).

Of the biochemical variables, lipid content and muscle TPP were inversely correlated with muscle THIAM and hepatic TotTh. PCA demonstrated that BPr and BS salmon shared a high lipid content and high individual *n*−*3* PUFA concentrations compared to GoF salmon. Alpha-linoleic acid (18:3*n*−*3*) was more strongly associated with BPr salmon than with BS salmon, and negatively associated with GoF salmon. However, DHA and docosapentaenoic acid (DPA*n*−*3*, 22:5*n*−*3*) were more strongly associated with BS salmon than BPr salmon, and even more clearly when running PCA with FAs alone (Supplementary Figure S7). Thus, the *n*−*3* PUFAs and PUFAs in total were most strongly associated with BS salmon. Furthermore, muscle TPP was associated with BS salmon (Figure 9). Muscle TPP thus had the strongest positive correlation with PUFAs, whereas muscle THIAM and hepatic TotTh were negatively correlated with PUFAs and lipids and were associated with GoF salmon. Hepatic MDA was associated with BS salmon and was positively correlated with PUFAs, whereas hepatic G6PDH was associated with GoF salmon and was negatively correlated with lipid content. PCA run with only biochemical variables also separated the salmon of the three areas and resulted in similar relationships to those including FAs in the analysis (Supplementary Figure S7).

Of the other FAs, a minor MUFA vaccenic acid (18:1*n*−*7*) and individual *n*−*6* PUFAs, eicosadienoic acid (20:2*n*−*6*), linoleic acid (18:2*n*−*6*), and arachidonic acid (ARA, 20:4*n*−*6*) were the strongest associated with BS salmon in PCA (Figure 9), and 18:1*n*−*7* was even stronger when PCA was run with FAs alone (Supplementary Figure S7). Of these FAs 18:1*n*−*7*, 18:2*n*−*6*, and ARA indicated eating herring, but 18:1*n*−*7* and 18:2*n*−*6* secondarily also eating in the BS. For BPr salmon, the association on the PC 2 axis with ARA was negative. The most prevalent MUFA, oleic acid (18:1*n*−*9*), was most strongly associated with BPr salmon, as was a minor MUFA, heptadecenoid acid (17:1*n*−*8*) (Figure 9). These both indicated feeding on sprat. The SFAs myristic acid (14:0), which indicates feeding in the southern Baltic Sea, and palmitic acid (16:0), the most common SFA, were associated with BPr salmon, whereas stearic acid (18:0) showed relative enrichment in the GoF salmon.

According to the PCA biplots, whether FAs were included or not, muscle carotenoids had a strong association with BPr salmon and not with GoF salmon. In contrast, hepatic EROD was associated with GoF salmon and not with BPr salmon (Figure 9).

## 4. Discussion

The differences in the FA composition and biochemical properties of prey fish between the three areas were reflected in the three salmon groups. The fatty acid composition of salmon indicated their primary prey fish [10], and the biochemical properties were related to the nutrient concentrations of prey fish in the areas. The amounts of lipids and PUFAs—more specifically of *n*−*3* PUFAs and even DHA that all accumulated in salmon according to the lipid content of prey fish—were reflected in the impaired thiamine status of salmon via the peroxidation of *n*−*3* PUFAs.

### 4.1. High G6PDH Activity Was Associated with Low Lipid Content

A high G6PDH activity in salmon liver was clearly associated with a low dietary and body lipid content. Consistent with results obtained experimentally with other fish species [53,54,55], a low dietary lipid content of prey fish in the GoF was thus reflected in the high activity of hepatic G6PDH [53,75].

The insignificant difference in the body total lipid content and the hepatic G6PDH activity between the salmon of the BPr and BS suggests that the supply of lipids from herring in the BS differed little from that of prey fish in the BPr. Apparently, herring as the main prey fish in the BS were approximately as lipid-rich as average sprat and herring in the prey fish biomass of salmon in the BPr [10]. According to Mikkonen et al. [17], herring have only formed a minor part of the total salmon food biomass in the BPr during the period 1994–2005. Salmon prey in the BPr has thus largely consisted of more lipid-rich prey fish, sprat, the dietary connection of which was consistently traced by FASA as described by Keinänen et al. [3]. FAs 18:1*n*−*9*, 17:1*n*−*8*, and 14:0 were typical for salmon from the BPr, and of these, the most common fatty acid in sprat, 18:1*n*−*9* and a minor MUFA 17:1*n*−*8* were the tracers of sprat [10,50], whereas clupeids have contained 14:0 in larger proportions in the southern parts of the Baltic Sea than in its northern areas [10,67]. However, M74 mortality was insignificant among the offspring of salmon females that ascended for spawning in the falls of 2002–2004 [4] after the size of the sprat stock had decreased to its lowest since the early 1990s [9,17].

The sprat caught from the BS were the study’s fattiest prey fish specimens. Sprat migrate to some extent to the BS from the more southern Baltic Sea, where they reproduce [41], but have comprised <5−10% of salmon prey fish biomass in the BS [17]. Accordingly, the FA signature of herring [10,67] was reflected in BS salmon, with larger proportions of 18:1*n*−*7*, i.e., the elongation product of 16:1*n*−*7*, and individual *n*−*6* PUFAs, as Keinänen et al. [3] also detected. These *n*−*6* and *n*−*7* FAs indicate that herring, contrary to solely plankton-feeding sprat [76,77,78,79], also feed on benthic invertebrates and food organisms with FAs of microalgae of freshwater origin [10,31,80].

### 4.2. Lipid Peroxidation Product MDA Was Associated with n−3 PUFAs

Although the lipid content of salmon from the BPr and BS did not differ significantly, the concentration of PUFAs and the concentration of *n*−*3* PUFAs in pairwise testing were significantly greater in BS salmon (50 ± 1 mg g^−1^) than in BPr salmon (42 ± 2 mg g^−1^). Consistently, the hepatic MDA concentration was largest in BS salmon, indicating a higher peroxidation rate of PUFAs [36] in them than in salmon of the other study areas. A strong positive correlation between the concentrations of MDA and PUFAs was an indication of their interdependence, whereas the MDA concentration was not correlated at all with the lipid content or the concentrations of the other FA classes. Specifically, *n*−*3* PUFAs, when oxidized, give rise to MDA [36,66]. The hepatic MDA concentration increased exponentially with an increase in the concentration of *n*−*3* PUFAs, i.e., on average 0.2 µmol MDA g^−1^ per a 1 mg g^−1^ increase in *n*−*3* PUFA (0.1 µmol MDA g^−1^ per a 1 mg g^−1^ increase in PUFA) in the present study. This was less than in salmon during their spawning migration and pre-spawning fasting, when an increase of 1% in the PUFA proportion was associated with an average increase of 1.6 μmol g^−1^ in hepatic MDA concentration [71]. A smaller and similar MDA concentration in salmon of the BPr and GoF, despite a larger concentration of *n*−*3* PUFAs in BPr salmon, may have arisen from the more favorable antioxidant status of BPr salmon compared to GoF salmon [81].

For its part, a larger concentration of *n*−*6* PUFAs in the dominant prey fish in the BS, herring, increased the concentration of total PUFAs in BS salmon. Although *n*−*6* PUFAs also produce some MDA, 4-hydroxy−2-nonenal is the principal product of *n*−*6* PUFA peroxidation [82,83,84]. In addition, *n*−*3* PUFAs amount to five times more than *n*−*6* PUFAs in salmon, and *n*−*3* PUFAs are, therefore, the principal source of MDA in Baltic salmon.

### 4.3. Thiamine Status Was Negatively Associated with Lipids and n−3 PUFAs

The concentration of TotTh in sprat and herring was similar and did not differ seasonally or spatially. The differences in the concentrations of hepatic TotTh and muscle thiamine components in salmon between the areas, therefore, arise from differences in the dietary lipid content and FA composition. The supply of thiamine per lipid and PUFA in the study year has been lowest among the actual prey fish biomass of salmon in the BS and highest in the GoF. Surprisingly, in the investigation by Keinänen et al. [9], in which the real prey biomasses of salmon was estimated by taking into account the abundance of each year class of sprat and herring, the thiamine concentration in the eggs of spawning salmon in the 1990s was smaller the larger the supply of thiamine and fat from sprat in the BPr in the preceding year. This is because the consumption of thiamine in metabolism increases with the increase in dietary energy [20,26]. This increases salmon’s need of thiamine, the dietary energy of which is largely obtained in the form of PUFAs that are prone to peroxidation, and thiamine is consumed in antioxidant action against the peroxidation [22,24]. This metabolic fact appeared in the present study as the smallest hepatic TotTh concentration in BS salmon with the highest hepatic MDA concentration, and as the largest hepatic TotTh concentration in GoF salmon with the lowest hepatic MDA concentration. The TotTh concentration of the liver has been shown to be a good indicator of the thiamine status and thiamine deficiency of salmon [71,85]. In the BPr salmon and BS salmon of the present study, the hepatic TotTh concentrations were roughly similar to those detected in the salmon of each area during the early spawning migration, when salmon were still feeding to some extent [71]. However, it decreased to one third by spawning from the start of the spawning run [71]. The same tendency in the thiamine status as in the liver could also be found in the muscle in the ratios of TotTh concentration per both the PUFA concentration and lipid content.

The effect of PUFAs on the thiamine status, which was stronger than the effect of lipids, was manifested in a significantly smaller hepatic TotTh concentration in BS salmon than in BPr salmon, whose lipid concentrations did not differ significantly. In addition, the hepatic TotTh concentration was not correlated with the SFA or MUFA content. There was no correlation either with the most common MUFA, 18:1*n*−*9*, which is characteristic of sprat [10,50] and was associated with BPr salmon in PCA, although it was the most common FA in salmon from all the study areas. On the contrary, an increase in the concentration of the most common PUFA, DHA, by 5 mg g^−1^ caused an approximately 17% decrease in the hepatic TotTh concentration of salmon.

PUFAs accumulated in BS salmon in large concentrations, because their principal prey fish, herring [17,43,44], had larger lipid contents there than in the other areas. In addition, the proportion of PUFAs of total FAs was or tended to be, according to Røjbek et al. [50] and Keinänen et al. [10], larger on average in herring than in sprat. The abundance of 1- and 2-year-old herring in the BS has increased the PUFA and *n*−*3* PUFA content of salmon, because the 2002 herring year class was exceptionally strong in the Gulf of Bothnia [38], and the largest concentrations of PUFAs and *n*−*3* PUFAs of herring were recorded in the youngest BS specimens in the present investigation. These, and the concentration of DHA, generally decreased with age for both sprat and herring, whereas the concentration of *n*−*6* PUFAs did not [10]. Preying abundantly on young herring has been significant for the body nutrient composition of these salmon caught late in the fall, because in the fall, the lipid and *n*−*3* PUFA content of herring in the BS was largest. In addition, in the fall, age 0 herring are already appropriate prey for salmon according to Salminen [86].

### 4.4. Thiamine Component Composition in Relation to Lipids and PUFAs

The proportion of TPP in salmon muscle and in the prey fish increased with the increase in lipid content, as was also found in pre-spawning salmon [71] and prey fish caught in the fall [51]. A similar association between muscle lipid content and TPP proportion appears to be found in five salmonine species of Lake Ontario [11]. However, the proportion of TPP increased somewhat more markedly with increasing PUFA concentration than with increasing body lipid content. Consistently, the proportion of TPP in muscle was larger in BS salmon (mean ± SE: 84 ± 1%, range: 83–86%) than in BPr salmon (64 ± 4%, 37–75%), whereas it was smallest in GoF salmon (47 ± 5%, 22–67%). After feeding in the Pacific Ocean, the proportion of TPP in the muscle in sockeye salmon (*Oncorhynchus nerka* Walbaum), 48 ± 6%, was similar to GoF salmon [87]. In BS salmon, the muscle TPP proportion was near those observed in the muscle of salmon at spawning (78–90%), and that of the single M74 female of M74 monitoring in 2004 was 89% [71].

As the proportion of TPP in muscle decreased and that of THIAM increased with decreasing lipid and PUFA content, the proportion of TMP in GoF salmon was relatively high, almost equal to that of TPP. Overall, muscle TMP concentration seemed to vary according to the concentration of THIAM and not to that of TPP, apparently because of the role of TMP as an essential intermediate in thiamine transport across cell membranes [21,88]. Smaller muscle TMP and THIAM proportions in BS salmon, therefore, reflected higher thiamine mitochondrial binding as TPP [24].

The coenzyme form TPP accounted for a major part of hepatic thiamine, as in soft tissues of vertebrates in general [21]. However, the TPP concentration was almost 10 times higher in the liver than in the muscle in GoF salmon, and nearly two times higher in BS salmon. Although the TotTh concentration was even several times larger in the liver than in the muscle of salmon (in the BPr 4.0, BS 2.2, and GoF 5.3-fold), most of thiamine is in muscle tissue, which is the largest tissue in fish. Because >90% of TPP in muscle is bound to the enzymes of energy metabolism in the mitochondria [24], the smallest muscle THIAM concentrations, which decreased with lipid and PUFA content and even DHA alone, were found in the fattiest salmon, i.e., BS salmon (0.059–0.088 nmol g^−1^). These were nearly as low as the muscle THIAM concentration (0.030 nmol g^−1^) of the single M74 female in 2004 [3]. In contrast, in BPr and GoF salmon with the smaller proportions of TPP, 4- and 13-times larger proportions were as THIAM (and, therefore, as reserve thiamine for additional needs) than in BS salmon. In sockeye salmon that had been feeding in the Pacific Ocean with a highly diverse food web [89], the proportion of muscle THIAM (30.3%, 3.7 nmol g^−1^) was even 28-times larger [87] than in BS salmon.

The role of thiamine in the FA and energy metabolism of prey fish was also evident, because the largest concentrations of thiamine derivative TTP occurred in the fattiest prey fish, which were sprat in the fall. Hence, the smallest TotTh concentration in the fattiest prey fish, as also observed in earlier investigations by Vuorinen et al. [51] and Keinänen et al. [9], may be related to the depletion of thiamine in their metabolism.

### 4.5. Lipid Peroxidation Impairs Thiamine Status

Because thiamine functions as a catalyst and critical cofactor in the form of TPP for all the enzymatic reactions that participate in oxidative metabolism producing ATP [19,90], thiamine deficiency can in general result from a high-calorie diet [20,21]. Considering the growth, the nutritional requirement of thiamine for salmonines is estimated to be approximately 0.3 nmol kJ^−1^ [26], the supply of which was exceeded in salmon prey fish, except for the very youngest herring in the BS and the very youngest sprat in the BPr. However, lipids and PUFAs accumulated as greater concentrations in salmon than in prey fish, and both lipid content and the concentration of DHA in salmon increased the more fattier prey fish they ate, as has been found with other fatty fish species and in other studies with Atlantic salmon [30,31,55,91,92]. Very large muscle lipid percentages (7.5–18.5%) were detected in salmon of M74 monitoring at spawning in the early 1990s when M74 was at its strongest, whereas the muscle lipid percentages were smaller (3.3–5.9%) in 2001–2016 [4]. In the BS, the larger PUFA concentration of herring and the abundance of young herring with the larger DHA concentration than in older herring [10] also increased the concentrations of *n*−*3* PUFAs and DHA in salmon (29 mg DHA g^−1^ in BS salmon vs. 24 and 13 mg DHA g^−1^ in BPr and GoF salmon). Thus, feeding on a diet with a smaller thiamine concentration per lipid and PUFA unit, the degree of lipid peroxidation that was clearly associated with *n*−*3* PUFAs, was also highest in BS salmon.

Salmonines store lipids as TAGs in the visceral cavity and muscle tissue during the feeding migration [31,32]. After their hydrolysis, liberated FAs can be used as an energy source not only during feeding, e.g., for growth and swimming, but also during pre-spawning fasting for oocyte formation in preparing for spawning [18]. Although free radicals and ROS are generated even in normal cell metabolism [56,66], peroxidation of PUFAs increases degradation of thiamine because of thiamine acting as a site-specific antioxidant in lipid peroxidation [22,24,37]. This was manifested by a large MDA concentration in BS salmon, whose thiamine reserves in relation to lipids and PUFAs were already minimal during feeding migration due to lipid peroxidation. However, because thiamine deficiency in itself increases oxidative stress [22,56], with the use of FAs of body lipids during pre-spawning fasting and exogenous vitellogenesis, thiamine is depleted in a downward spiral due to the peroxidation of *n*−*3* PUFAs. There is, thus, too little thiamine in the tissues of a female salmon to be transported to the developing oocytes to be sufficient for the offspring until the end of the yolk-sac phase and starting external feeding [71]. This was found for a single M74 female in the fall of 2004, which, according to FASA (Supplementary Figure S1) and the ratio of the concentration of PCDD/Fs to the concentration of CoPCBs, had been eating herring in the BS [2,3,52]. In all the other salmon included then in M74 monitoring, the muscle FA signatures and the ratio of organochlorines were characteristic of those salmon feeding in the Baltic Proper.

Other factors, such as environmental toxicants, may increase general oxidative stress [65], but they did not impair the thiamine status of salmon. On the contrary, the hepatic TotTh concentration was largest in GoF salmon with the largest concentrations of PCBs and PCDDs [49] and, therefore, also the highest hepatic EROD activity.

### 4.6. The Least Carotenoids from the Leanest Prey Fish

The differences in the concentration of antioxidant carotenoids of prey fish between the study areas were clearly apparent in the muscle of salmon; the carotenoid concentration was largest in the BPr, and it was smallest in the GoF, i.e., the leanest salmon. The carotenoid concentration of salmon, therefore, had a positive correlation with the lipid percentage and PUFA concentration. The poor nutritional condition of prey fish in the GoF could be seen as a smaller carotenoid concentration in the muscle of GoF salmon than in the salmon of the other two areas, because carotenoids are transferred to salmon directly from crustaceans in the stomach of sprat and herring [59]. The nutrient content of prey fish was analyzed in whole fish, including the entrails, and the results for prey fish thus represented the actual diet of salmon.

Contrary to the present study, the carotenoid concentration was significantly larger in the muscle of pre-spawning salmon caught in the BS than in salmon caught in the BPr [71]. Carotenoid concentrations of salmon muscle appeared to vary greatly between the sea areas during the spawning migration [71], probably because many factors affected their concentrations in the lower food web [93]. In addition, the total carotenoid concentration of salmon muscle did not show any trend during the spawning migration and had no relationships with the concentration of TotTh of the ovaries, liver, or muscle, although at spawning in the river, it was much smaller than in salmon caught in the sea [71]. As the concentrations of organochlorines in salmon and their prey fish were significantly larger in the GoF than in the other areas [49], carotenoids may also have decreased while functioning as antioxidants against general oxidative stress caused by them [65]. Despite their large organochlorine concentrations [2,49] and high general oxidative stress [62,81], the thiamine status of GoF salmon was good based on the hepatic TotTh and muscle THIAM concentrations, although not as good as in sockeye salmon that had been feeding in the Pacific Ocean [87]. In addition, the GoF salmon have suffered from milder M74 than salmon feeding in the BPr [4]. This also shows that xenobiotic induced oxidative stress does not impair thiamine status; it is lipid peroxidation that impairs it.

### 4.7. EROD Was Associated with PCBs and PCDDs, but MDA and Thiamine Were Not

In addition to nutritional composition and biochemistry, the leanness of prey fish was reflected in the concentrations of organochlorines of salmon [49] that were caught during the feeding migration immediately after the intense annual growth period of fish [94]. A slow growth rate of the prey fish in the GoF is probably the most important reason for the largest concentrations of PCBs and PCDDs in GoF salmon compared to salmon from the other areas [49]. This is because organochlorines bioaccumulate according to age, and due to the retarded growth of prey fish in the GoF, salmon had consumed older fish there on average [49]. Moreover, the GoF possibly receives an extra loading of dioxins from the bottom sediments of the River Kymijoki [2,95].

According to Whyte et al. [64], structural analogues to the most toxic of PCDDs, TCDD, are the most potent inducers of EROD activity in fish. In the present study, the positive correlation of EROD activity was highly significant and strong with the concentrations of PCBs and PCDDs on a fresh weight basis. High EROD activity in salmon caught in the GoF during the late fall and winter of 2006 was also detected by Vuori et al. [62]. EROD activities in salmon from the BS and BPr did not differ, although the concentrations of PCBs and PCDDs were larger in BS salmon than in BPr salmon [49]. Because the concentrations of CoPCBs (the most toxic TCDD analogues of PCBs) are larger in sprat than in herring [49], and sprat is the principal prey fish of salmon in the BPr, unlike in the BS [17,39], the concentrations of CoPCBs were larger in BPr salmon than in BS salmon [49]. Due to the combined effect of organochlorine quantity and structure, the total WHO-TEQ therefore did not differ between the salmon of these two areas. Evidently, the organochlorines induced EROD activity, but they had no association with the concentrations of MDA or hepatic TotTh.

The positive correlations of some PCDD/Fs and PCBs with the hepatic G6PDH activity were apparently due only to the fact that both were largest in GoF salmon. In spite of possible oxidative stress due to xenobiotics [62,65], induced G6PDH activity probably resulted only from lipogenic activity [53,75], because the other NADPH-generating system, the pentose phosphate shunt with thiamine-dependent transketolase [24], was evidently functioning well, based on the good thiamine status of GoF salmon.

## 5. Conclusions

Salmon from the three areas of the Baltic Sea were separated similarly based on the FASA and the biochemical parameters that reflected the nutritional characteristics of the prey fish of each area. The high lipid and *n*−*3* PUFA content in prey fish and the abundance of such prey increased their concentrations in salmon. The increase in *n*−*3* PUFAs, and, in particular, DHA with the largest number of double bonds, caused exponential production of the peroxidation product MDA in the liver and impaired thiamine status in salmon tissues, for which the hepatic TotTh concentration appeared to be a sensitive indicator. Apparently, hepatic G6PDH activity was negatively associated with the high lipid content due to its suppression by dietary lipids. The increase in dietary and body lipids as the principal energy source for salmon increases the requirement of thiamine seen in their ratio and in proportions of thiamine components. Although the proportion of TPP, the coenzyme of the key enzymes in energy metabolism, increased in salmon muscle, and similarly in prey fish, with increasing lipid content, the concentration of the reserve thiamine component THIAM decreased. Thus, the present study demonstrated that the thiamine status of salmon can already be impaired, at least to some extent, during their feeding migration, and on the other hand, it ensured that the impaired thiamine status of salmon was not associated with organochlorines or general oxidative stress. In years when young herring in the BS are abundant and fatty, some cases of thiamine deficiency M74 may, therefore, result from feeding there. However, a comparatively small herring prey biomass in the BS cannot support numerous salmon compared to the BPr, where most salmon migrate to feed, and from where most M74 originate as a result of feeding abundantly on sprat. A worrying M74 situation can therefore be anticipated when the sprat stock is strong and young sprat are abundant, while the cod stock is small in the BPr, and deteriorates further when young herring are also abundant and fatty in the BS. Hence, it is not a particular prey species, but overall, an abundant intake of fish lipids containing long-chain *n*−*3* PUFAs and specifically DHA in high percentages, that can cause thiamine deficiency in salmonines and in fatty fish in general.

## Figures and Tables

**Table 2:** Mean (±SE) concentrations of thiamine pyrophosphate (TPP), thiamine monophosphate (TMP), free thiamine (THIAM), and total thiamine (TotTh) in the muscle and liver of salmon (*Salmo salar*) caught in the Baltic Proper (BPr), Bothnian Sea (BS), and Gulf of Finland (GoF) of the Baltic Sea in the late fall during feeding migration. *N* = number of observations.

TPP nmol g^−1^				TMP nmol g^−1^			THIAM nmol g^−1^			TotTh nmol g^−1^			*N*
Muscle													
BPr	4.530	±	0.366 ^a^	2.352	±	0.286 ^b^	0.322	±	0.149 ^a^	7.204	±	0.549	9
BS	6.581	±	1.032 ^b^	1.196	±	0.226 ^a^	0.075	±	0.005 ^a^	7.851	±	1.255	5
GoF	3.302	±	0.439 ^a^	2.626	±	0.241 ^b^	1.028	±	0.177 ^b^	6.956	±	0.528	10
Liver													
BPr	21.926	±	1.676 ^b^	6.694	±	0.698 ^b^	0.129	±	0.053 ^a^	28.748	±	2.264 ^b^	9
BS	12.236	±	1.519 ^a^	4.220	±	0.475 ^a^	0.557	±	0.062 ^b^	17.014	±	2.024 ^a^	5
GoF	31.914	±	1.942 ^c^	8.069	±	0.541 ^b^	0.137	±	0.091 ^a^	40.120	±	1.892 ^c^	5

A different letter as a superscript to SE indicates a significant difference (*p* < 0.05, Student Neuman–Kuels post hoc test) between the means of the three sea areas.

**Table 3:** Pearson correlations of the concentrations (nmol g^−1^) of thiamine pyrophosphate (TPP), thiamine monophosphate (TMP), unphosphorylated or free thiamine (THIAM), and total thiamine (ToTh), and the proportions of TPP and THIAM in salmon (*Salmo salar*) muscle and in sprat (*Sprattus sprattus*) and herring (*Clupea harengus*) with the lipid content (%) and the concentrations (i.e., lipid-weighed proportions, mg g^−1^) of fatty acid classes of salmon. Salmon and prey fish caught in the three Baltic Sea areas: the Baltic Proper; the Bothnian Sea; and the Gulf of Finland.

		TPP	TMP	THIAM	TotTh	TPP%	THIAM%
Salmon	Body lipid	0.458	−0.583	−0.662	−0.203	0.680	−0.664
		0.024	0.003	0.0004	0.341	0.0003	0.0004
	SFA	0.378	−0.595	−0.656	−0.277	0.624	−0.624
		0.069	0.002	0.001	0.190	0.001	0.001
	MUFA	0.445	−0.551	−0.595	−0.171	0.628	−0.584
		0.029	0.005	0.002	0.425	0.001	0.003
	PUFA	0.503	−0.553	−0.644	−0.140	0.706	−0.680
		0.012	0.005	0.001	0.515	0.0001	0.0003
	*n*−*3* PUFA	0.490	−0.541	−0.635	−0.140	0.690	−0.671
		0.015	0.006	0.001	0.514	0.0002	0.0003
	*n*−*6* PUFA	0.574	−0.611	−0.673	−0.125	0.784	−0.715
		0.003	0.002	0.0003	0.562	<0.0001	<0.0001
Sprat	Body lipid	0.453	0.447	−0.488	−0.193	0.754	−0.755
		0.068	0.072	0.047	0.458	0.001	0.001
Herring	Body lipid	0.584	0.599	−0.339	−0.172	0.635	−0.733
		0.003	0.002	0.106	0.420	0.001	<0.0001

Correlation coefficients and the *p*-value below are given; salmon and prey fish lipid content (%) was measured in the total body homogenate; fatty acid concentrations were measured in the muscle of salmon, and in the homogenized whole-body pools in sprat and herring; the number of sprat and herring pools (each composed of 7–133 individual fish) was 17 and 24, respectively, and the number of salmon was 24; the lipid content and the concentrations of FA classes of sprat and herring were derived from Keinänen et al. [10].

**Table 4:** Pearson correlations of hepatic glucose 6-phosphate dehydrogenase activity (G6PDH, µmol min^−1^ mg prot^−1^) and malondialdehyde (MDA, µmol g^−1^) and total thiamine (TotTh, nmol g^−1^) concentrations with whole-body lipid content (%) and the concentrations (i.e., lipid-weighed proportions, mg g^−1^) of the fatty acid classes of salmon (*Salmo salar*) caught in the Baltic Proper, the Bothnian Sea, and the Gulf of Finland in the late fall.

	G6PDH	MDA	TotTh
Lipid	−0.625	0.319	−0.537
	0.004	0.183	0.018
SFA	−0.440	0.106	−0.299
	0.059	0.667	0.213
MUFA	−0.480	0.216	−0.448
	0.038	0.374	0.054
*n*−*3* PUFA	−0.719	0.526	−0.681
	0.001	0.021	0.001
*n*−*6* PUFA	−0.774	0.594	−0.758
	0.0001	0.007	0.0002
PUFA	−0.732	0.534	−0.695
	0.0004	0.019	0.001

Correlation coefficients and the *p*-value below are given; the total lipid contents were derived from Vuorinen et al. [49]; the number of salmon was 19.

## Data Availability

The datasets used and analyzed during the current study are available from the corresponding author on reasonable request.

## Supplementary Materials

The following supporting information can be downloaded at: https://www.mdpi.com/article/10.3390/biom12040526/s1. Figure S1: FA-based PCA biplot of female salmon included in M74 monitoring in 2004; Figure S2: total lipid, carotenoids, and thiamine in sprat, herring, and salmon; Figure S3: SFAs, MUFAs, and PUFAs in sprat, herring, and salmon; Figure S4: ratios of TotTh to lipid and PUFA in sprat, herring, and salmon; Figure S5: whole-body concentrations of TotTh and thiamine components and their proportions of TotTh in sprat and herring in the fall and spring; Figure S6: hepatic and muscle thiamine component concentrations and proportions in salmon; Figure S7: PCA biplots, based on FAs and biochemical indices, of salmon during feeding migration; Table S1: prey fish pool description; Table S2: the identified Fas; Table S3: characteristics of sprat and herring; Table S4: the effects of species, feeding area, and season on parameters of the prey fish by MANOVA and LsMeans.
