# Supplementary material for: High Lipid Content of Prey Fish and n−3 PUFA Peroxidation Impair the Thiamine Status of Feeding-Migrating Atlantic Salmon (Salmo salar) and Is Reflected in Hepatic Biochemical Indices
Source: Biomolecules. 2022 Mar 30;12(4):526. doi: 10.3390/biom12040526 (PMC9031544; doi:10.3390/biom12040526)
Supplement: Supplementary file 1 [file biomolecules-12-00526-s001.zip › biomolecules-1641503-supp update.pdf]

Supplementary material

Marja Keinänen, Soili Nikonen, Reijo Käkelä, Tiina Ritvanen, Mervi Rokka, Timo Myllylä, Jukka Pönni, Pekka J. Vuorinen: High lipid content of prey fish and n-3 PUFA peroxidation impair the thiamine status of feeding-migrating Atlantic salmon (*Salmo salar*) and is reflected in hepatic biochemical indices. *Biomolecules* **2022**, *12*, 526. https://doi.org/10.3390/biom12040526

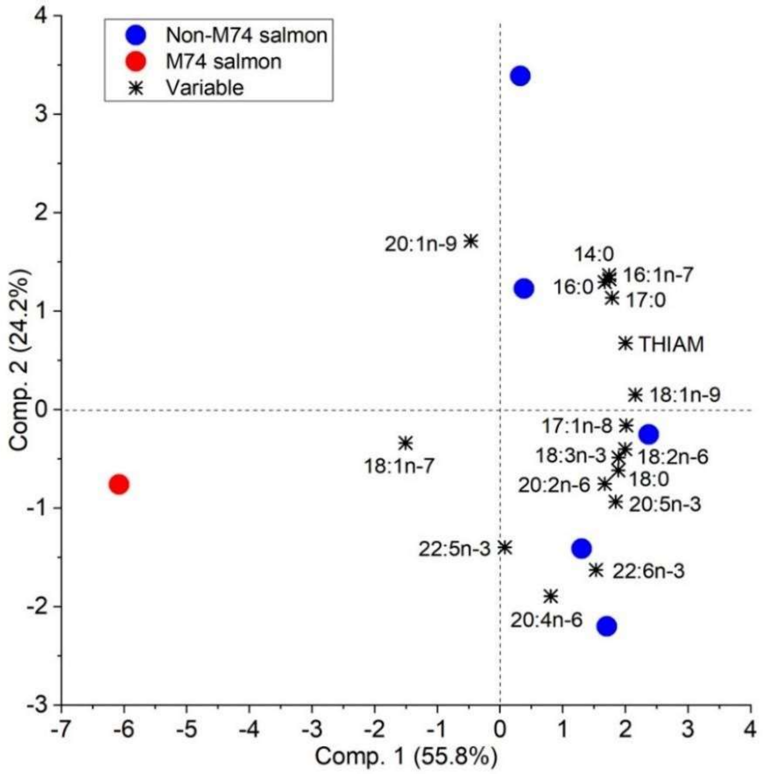

Supplementary Figure S1. Biplot from the PCA model with principal components 1 and 2 for salmon (*Salmo salar*) female spawners from the M74-monitoring year 2004 (classified as females with or without M74), and variables measured: the concentrations of individual fatty acids in muscle and that of THIAM (= unphosphorylated or free thiamine) in eggs. Data derived from Keinänen et al. [3].

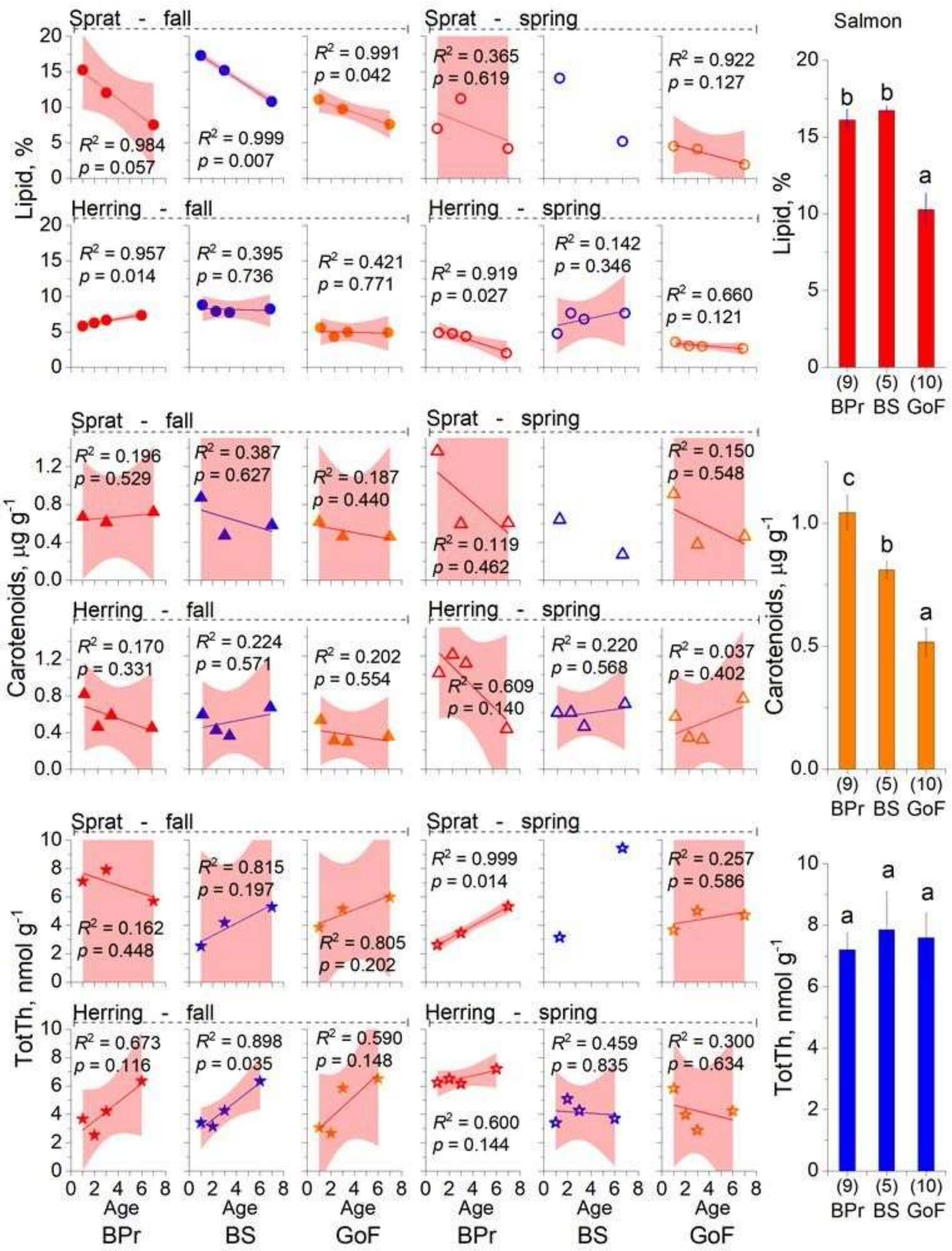

Supplementary Figure S2. Total lipid content in the whole body of sprat (*Sprattus sprattus*) and herring (*Clupea harengus*) in the fall and spring according to age and in muscle of salmon (*Salmo salar*) (right column, mean ± SE, in the late fall), and the concentrations of total carotenoids and total thiamine (TotTh) in sprat and herring and in the muscle of salmon, in the three areas of the Baltic Sea, the Baltic Proper (BPr) the Bothnian Sea (BS), and the Gulf of Finland (GoF). For the sprat and herring, linear regressions with 95% confidence bands and coefficients of determination (R^2) and significance (p) of the models are given. Different letters denote significant ($P < 0.05$) differences between the areas in salmon. The number of salmon is given in parentheses; sprat and herring pools were each composed of 7–133 individual fish. Body lipid contents of sprat and herring were derived from Keinänen et al. [10] and of salmon from Vuorinen et al. [49].

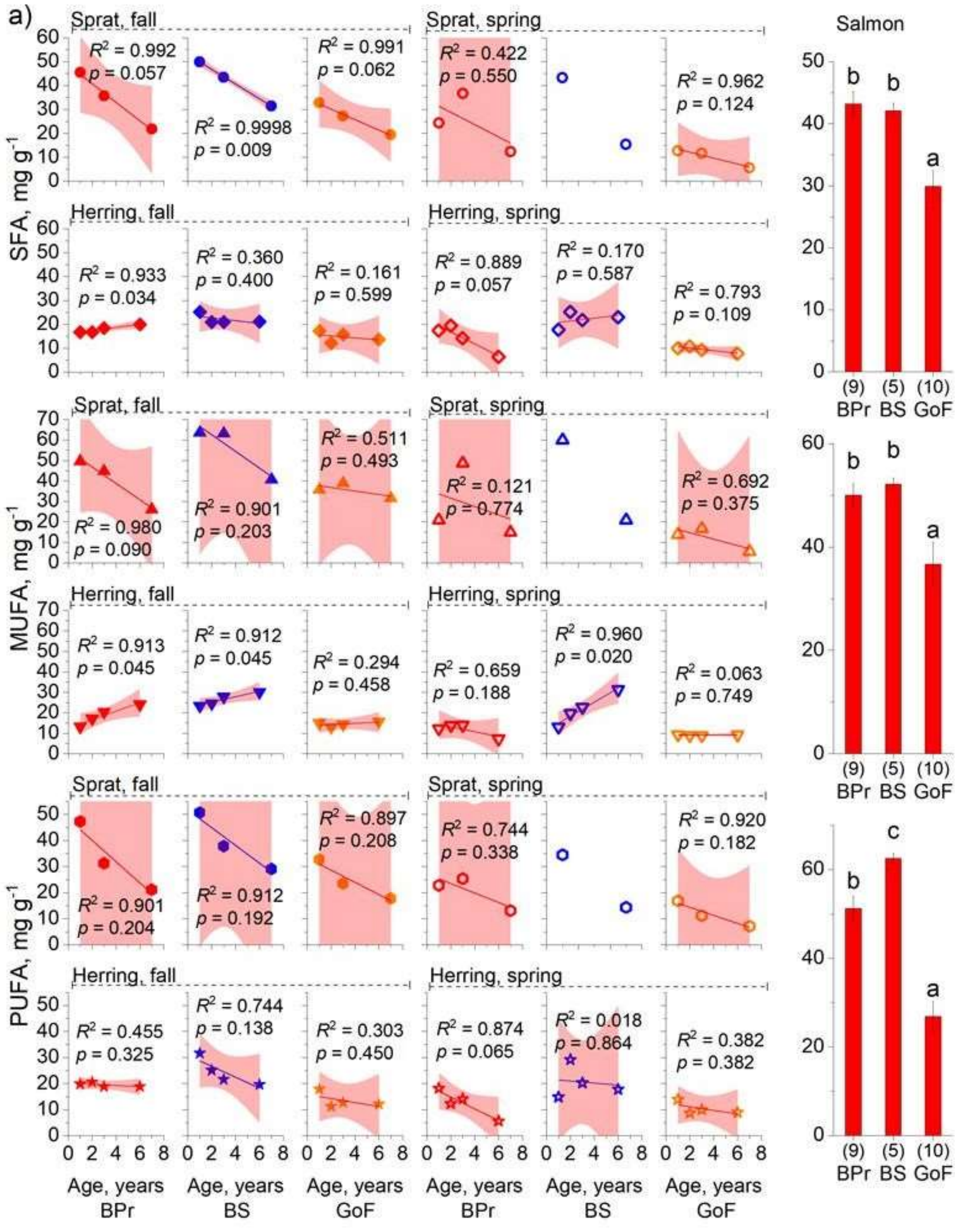

a)
Sprat, fall
Sprat, spring
Herring, fall
Herring, spring
Salmon
SFA, mg g-1
MUFA, mg g-1
PUFA, mg g-1
R2 = 0.992
p = 0.057
R2 = 0.9998
p = 0.009
R2 = 0.991
p = 0.062
R2 = 0.422
p = 0.550
R2 = 0.962
p = 0.124
R2 = 0.933
p = 0.034
R2 = 0.360
p = 0.400
R2 = 0.161
p = 0.599
R2 = 0.889
p = 0.057
R2 = 0.170
p = 0.587
R2 = 0.793
p = 0.109
R2 = 0.980
p = 0.090
R2 = 0.901
p = 0.203
R2 = 0.511
p = 0.493
R2 = 0.121
p = 0.774
R2 = 0.692
p = 0.375
R2 = 0.913
p = 0.045
R2 = 0.912
p = 0.045
R2 = 0.294
p = 0.458
R2 = 0.659
p = 0.188
R2 = 0.960
p = 0.020
R2 = 0.063
p = 0.749
R2 = 0.901
p = 0.204
R2 = 0.912
p = 0.192
R2 = 0.897
p = 0.208
R2 = 0.744
p = 0.338
R2 = 0.920
p = 0.182
R2 = 0.455
p = 0.325
R2 = 0.744
p = 0.138
R2 = 0.303
p = 0.450
R2 = 0.874
p = 0.065
R2 = 0.018
p = 0.864
R2 = 0.382
p = 0.382
Age, years
BPr
BS
GoF
(9) (5) (10)
BPr BS GoF
b b a
b b a
b c a

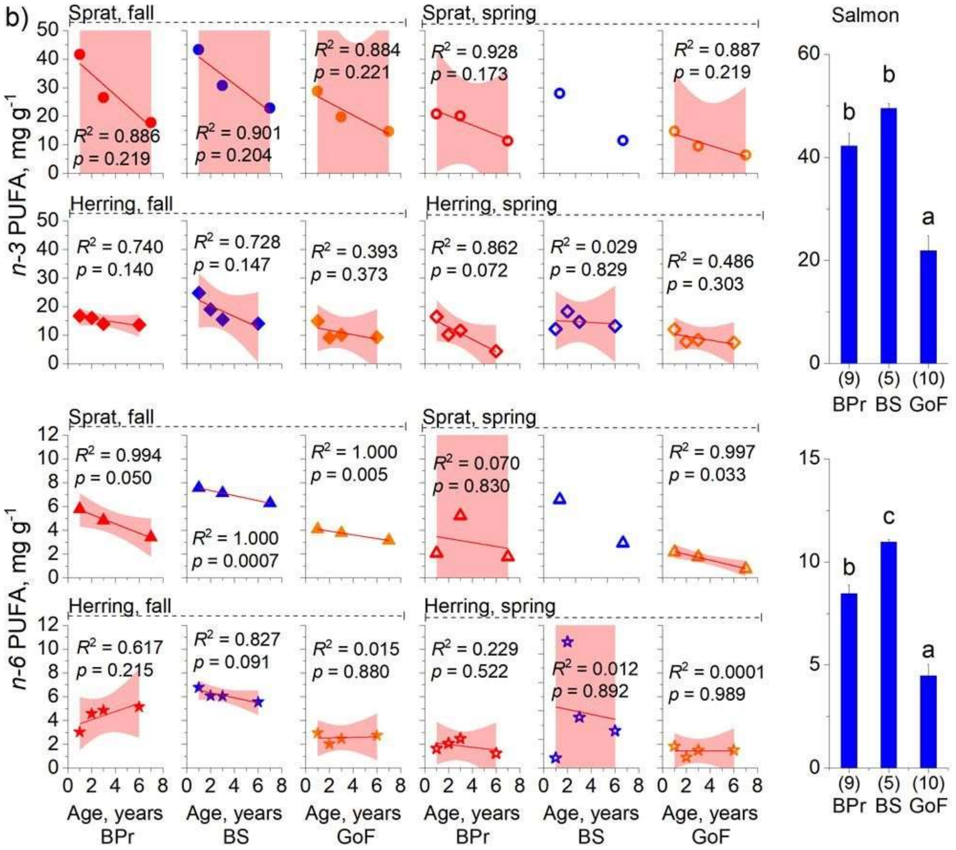

Supplementary Figure S3. Concentrations of a) SFAs, MUFAs, and PUFAs and b) *n-3* PUFAs and *n-6* PUFAs in the whole-body of sprat (*Sprattus sprattus*) and herring (*Clupea harengus*) in the fall and spring according to age, and in salmon (*Salmo salar*) (right column, mean ± SE, in the late fall), in the three areas of the Baltic Sea, the Baltic Proper (BPr), the Bothnian Sea (BS), and the Gulf of Finland (GoF). For the sprat and herring, linear regressions with 95% confidence bands and coefficients of determination (R^2) and significance (p) of the models are given. Different letters denote significant ($P < 0.05$) differences in salmon between the areas. The number of salmon is given in parentheses. The individual pools of sprat and herring were composed of 7–133 fish specimens. The concentrations of FAs of sprat and herring were derived from Keinänen et al. [10].

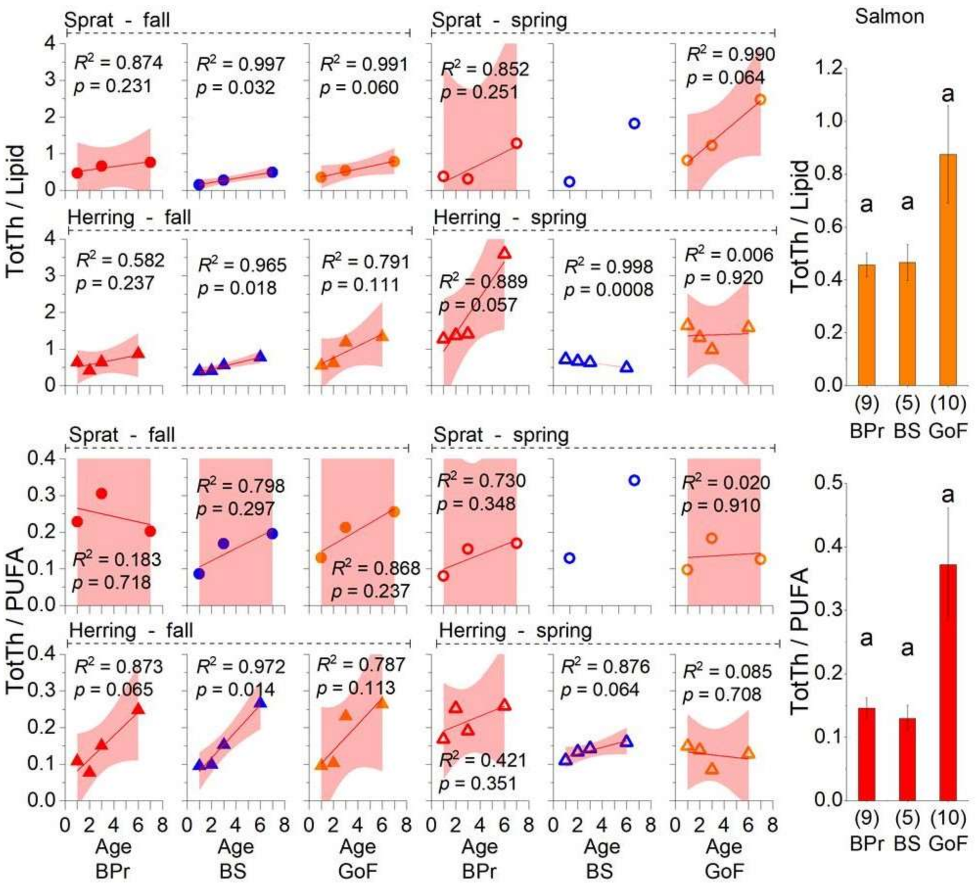

Supplementary Figure S4. Ratios of the total thiamine to the whole-body lipid (TotTh/Lipid) and PUFAs (TotTh/PUFA) in sprat (*Sprattus sprattus*) and herring (*Clupea harengus*) in the fall and spring according to age, and in the muscle of salmon (*Salmo salar*) (right column, mean ± SE, in the late fall), from the three areas of the Baltic Sea, the Baltic Proper (BPr), the Bothnian Sea (BS), and the Gulf of Finland (GoF). For the sprat and herring, linear regressions with 95% confidence bands and coefficients of determination (R^2) and significance (p) of the models are given. Different letters denote significant ($P < 0.05$) differences in salmon between the areas. The number of salmon is given in parentheses. The individual pools of sprat and herring were composed of 7–133 fish specimens. The lipid contents and concentrations of PUFAs of sprat and herring were derived from Keinänen et al. [10].

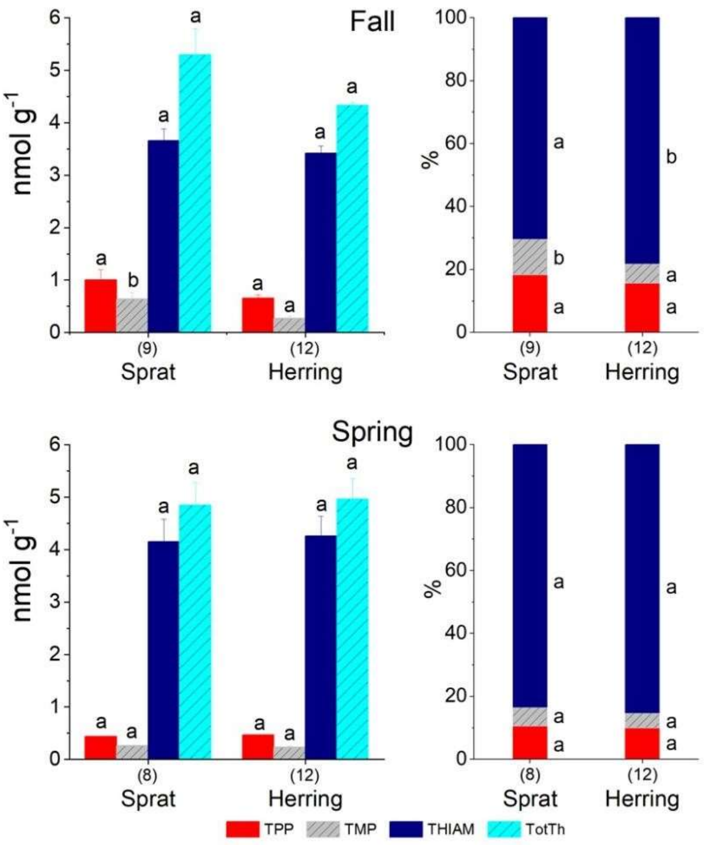

Supplementary Figure S5. Mean (± SE) concentrations (left column) of thiamine pyrophosphate (TPP), thiamine monophosphate (TMP), unphosphorylated or free thiamine (THIAM) and total thiamine (TotTh), and the proportions (right column) of TPP, TMP, and THIAM of TotTh in the whole body of sprat (*Sprattus sprattus*) and herring (*Clupea harengus*) from the Baltic Proper, the Bothnian Sea, and the Gulf of Finland in the fall and spring. Different letters denote statistically significant ($P < 0.05$) differences between the species. The number of sprat and herring pools is given in parentheses. For sprat, each pool was composed of 12–92 and for herring of 7–133 specimens.

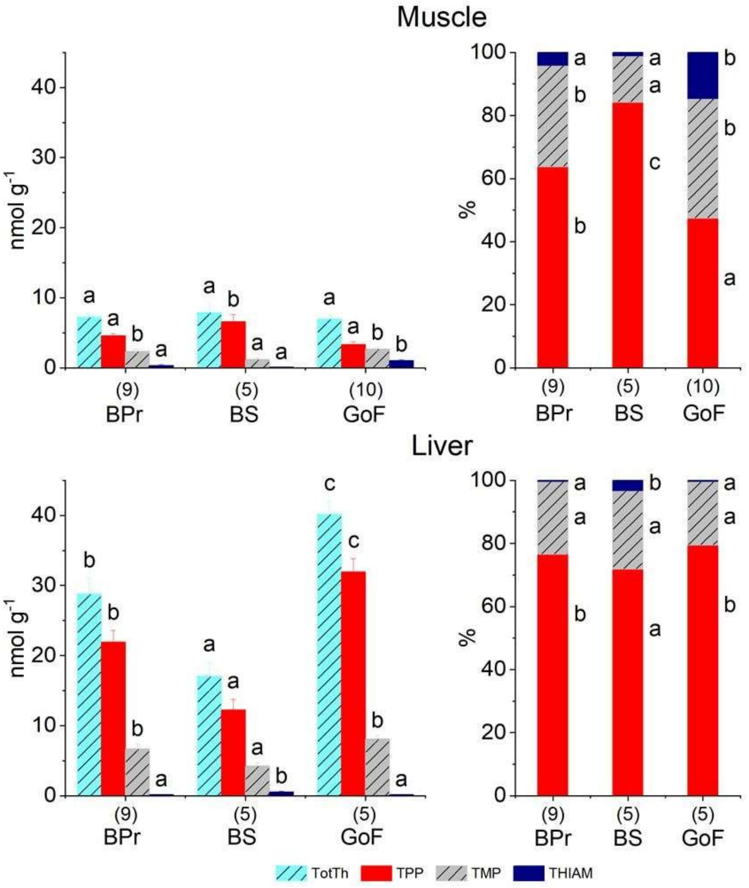

Supplementary Figure S6. Mean (± SE) concentrations (left columns) of thiamine pyrophosphate (TPP), thiamine monophosphate (TMP), and unphosphorylated or free thiamine (THIAM), and the proportions (right column) of TPP, TMP, and THIAM in the muscle (upper row) and liver (lower row) of salmon (*Salmo salar*) from the Baltic Proper (BPr), the Bothnian Sea (BS), and the Gulf of Finland (GoF) in the fall. Different letters denote statistically significant ($P < 0.05$) differences between the areas. The number of salmon is given in parentheses.

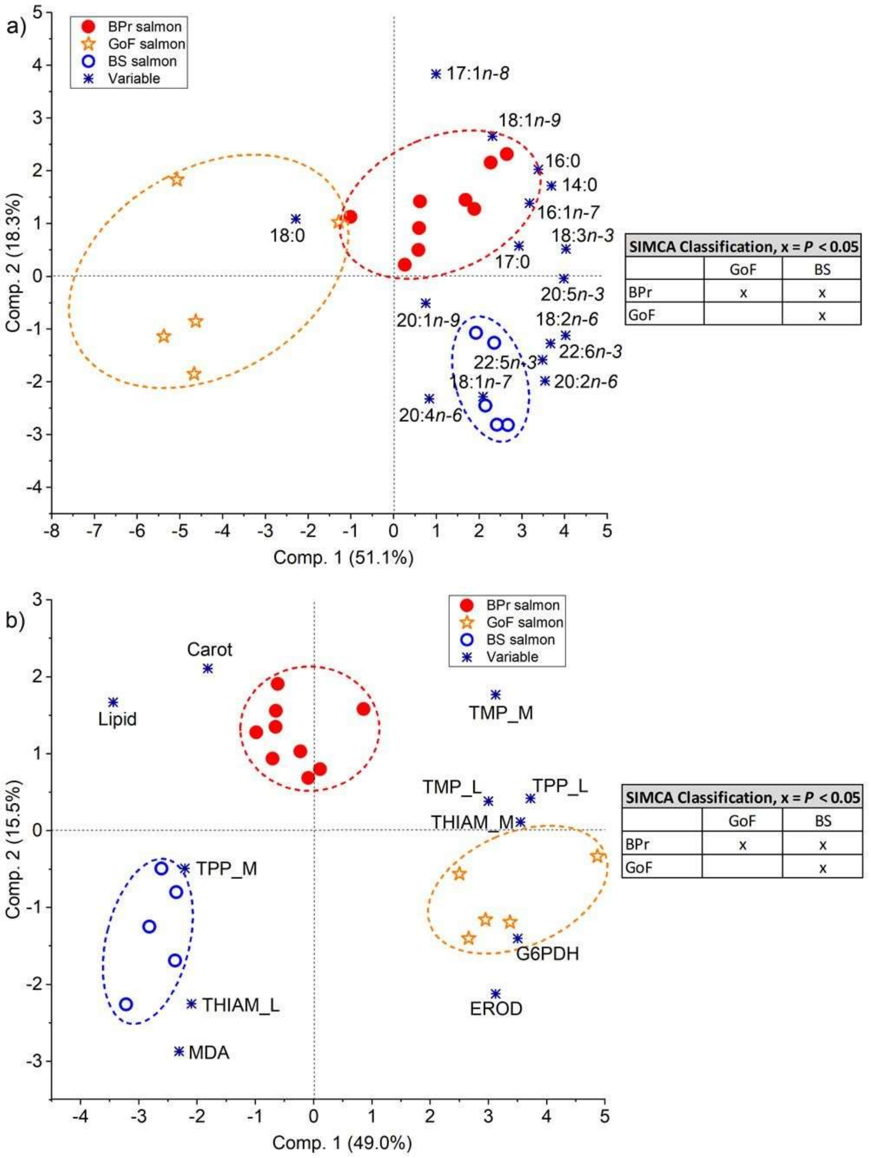

SIMCA Classification, x = $P < 0.05$ (a)

	GoF	BS
BPr	x	x
GoF		x

SIMCA Classification, x = $P < 0.05$ (b)

	GoF	BS
BPr	x	x
GoF		x

Supplementary Figure S7. Biplots from the PCA model with principal components 1 and 2 for salmon (*Salmo salar*) caught in the Baltic Proper (BPr), the Bothnian Sea (BS), and the Gulf of Finland (GoF) in the late fall: a) the concentrations of individual fatty acids and b) various biochemical indices: the activity of glucose 6-phosphate dehydrogenase (G6PDH) and 7-ethoxyresorufin-*O*-deethylase (EROD) and the concentration of malondialdehyde (MDA) in the liver, the concentrations of thiamine components (TPP = thiamine pyrophosphate, TMP = thiamine monophosphate, and THIAM = unphosphorylated or free thiamine) in the muscle (M) and liver (L), the concentration of total carotenoids (Carot) in the muscle, and the whole-body lipid content (Lipid). Results of the paired SIMCA tests for salmon from the three Baltic Sea areas are also presented.

Supplementary Table S1. Composition of sprat (*Sprattus sprattus*) and herring (*Clupea harengus*) in pools: age in years, number of specimens, mean weight, mean length, and total weight of a pool in the fall (F) and spring (S) from the three study areas BPr (Baltic Proper), BS (Bothnian Sea), and GoF (Gulf of Finland).

Species	Season	Area	Age	Number ofspecimens	Mean weight g	Mean length mm	Weight of pool g
Sprat	F	BPr	1	31	6.3	101.2	196
Sprat	F	BPr	3	13	8.9	115.4	116
Sprat	F	BPr	7	12	10.9	126.4	131
Sprat	F	BS	1	20	8.0	105.2	160
Sprat	F	BS	3	17	10.5	119.7	178
Sprat	F	BS	7	28	12.0	127.0	335
Sprat	F	GOF	1	34	5.6	96.5	189
Sprat	F	GOF	3	36	8.6	116.7	311
Sprat	F	GOF	7	33	10.1	126.0	333
Herring	F	BPr	1	93	11.5	121.5	1067
Herring	F	BPr	2	74	19.2	145.1	1421
Herring	F	BPr	3	86	27.9	162.6	2397
Herring	F	BPr	6	11	42.3	183.0	465
Herring	F	BS	1	67	11.3	121.9	760
Herring	F	BS	2	50	19.4	146.2	969
Herring	F	BS	3	36	26.3	162.7	948
Herring	F	BS	6	17	35.9	180.1	611
Herring	F	GOF	1	74	7.7	110.4	568
Herring	F	GOF	2	57	12.5	133.5	711
Herring	F	GOF	3	25	18.1	149.1	453
Herring	F	GOF	6	8	22.0	159.9	176
Sprat	S	BPr	1	15	2.4	79.0	36
Sprat	S	BPr	3	92	8.5	114.1	783
Sprat	S	BPr	7	39	10.1	126.2	392
Sprat	S	BS	1	Not caught			
Sprat	S	BS	3	46	8.5	111.6	392
Sprat	S	BS	7	25	11.4	129.8	284
Sprat	S	GOF	1	19	3.5	90.5	66
Sprat	S	GOF	3	61	7.6	113.7	465
Sprat	S	GOF	7	23	9.5	128.5	219
Herring	S	BPr	1	13	5.3	99.6	69
Herring	S	BPr	2	133	9.9	120.2	1314
Herring	S	BPr	3	100	17.7	148.9	1767
Herring	S	BPr	6	24	31.5	176.4	757
Herring	S	BS	1	38	4.3	90.1	163
Herring	S	BS	2	88	11.0	123.7	967
Herring	S	BS	3	63	19.6	151.3	1233
Herring	S	BS	6	14	31.6	176.7	443
Herring	S	GOF	1	7	2.7	82.7	19
Herring	S	GOF	2	133	6.1	105.3	805
Herring	S	GOF	3	52	14.1	142.2	733
Herring	S	GOF	6	17	25.4	170.4	432

Supplementary Table S2. The identified fatty acids (FAs) with the abbreviation and common names. The 16 FAs, the mean proportion of which was >0.4%, and which were included in calculations, are also indicated.

Abbreviation	Common name	Included in the 16 FAs
14:0	myristic acid	x
14:1*n-5*	myristoleic acid	
15:0	pentadecanoic acid	
16:0	palmitic acid	x
16:1*n-7*	palmitoleic acid	x
17:0	heptadecanoic acid	x
17:1*n-8*	heptadecenoic acid	x
18:0	stearic acid	x
18:1*n-9*	oleic acid	x
18:1*n-7*	vaccenic acid	x
18:2*n-6*	linoleic acid	x
18:3*n-3*	alpha-linolenic acid	x
18:4*n-3*	stearidonic acid	
20:0	arachidic acid	
20:1*n-9*	gondoic acid	x
20:2*n-6*	eicosadienoic acid	x
20:3*n-3*	eicosatrienoic acid	
20:4*n-6*	arachidonic acid (ARA)	x
20:5*n-3*	eicosapentaenoic acid (EPA)	x
22:5*n-3*	docosapentaenoic acid (DPA)	x
22:6*n-3*	docosahexaenoic acid (DHA)	x

Supplementary Table S3. The mean and range (minimum and maximum) of the body weight, total length, and condition factor (CF) of sprat (*Sprattus sprattus*) and herring (*Clupea harengus*), and (on a wet weight basis) the body lipid content and concentrations of thiamine pyrophosphate (TPP), thiamine monophosphate (TMP), unphosphorylated or free thiamine (THIAM), total thiamine (TotTh), TotTh per total lipid and PUFAs and total carotenoids, as well as concentrations of the fatty acid classes in sprat and herring from the Baltic Proper (BPr), the Bothnian Sea (BS), and the Gulf of Finland (GoF).

	BPr			BS			GoF		
Variable	Mean	Range	*N*	Mean	Range	*N*	Mean	Range	*N*
Sprat									
Weight, g	7.9	2.4–10.9	6	10.1	8.0–12.0	5	7.5	3.5–10.1	6
Length, mm	110	79–126	6	119	105–130	5	112	90–129	6
CF	0.55	0.49–0.61	6	0.60	0.52–0.69	5	0.52	0.45–0.62	6
Total lipid, %	9.5^{B}	4.17–15.2	6	12.5^{B}	5.19–17.3	5	6.5^{A}	1.91–11.1	6
TPP, nmol g^{-1}	1.052	0.263–2.334	6	0.608	0.400–0.877	5	0.526	0.355–0.639	6
TMP, nmol g^{-1}	0.658^{A}	0.148–1.492	6	0.336	0.259–0.424	5	0.364^{B}	0.149–0.612	6
THIAM, nmol g^{-1}	3.642	2.207–4.657	6	3.971	1.709–8.713	5	3.835	2.682–5.057	6
TotTh, nmol g^{-1}	5.352	2.618–7.912	6	4.914	2.519–9.434	5	4.725	3.672–5.968	6
TotTh/lipid	0.64	0.31–1.28	6	0.59	0.16–1.82	5	1.03	0.35–2.48	6
TotTh/PUFA	0.22	0.11–0.41	6	0.22	0.05–0.66	5	0.33	0.12–0.66	6
Total carotenoids, mg g^{-1}	0.76	0.60–1.36	6	0.57	0.27–0.87	5	0.55	0.38–0.912	6
SFA, mg g^{-1}	29.4^{B}	12.4–45.5	6	36.7^{B}	15.3–49.9	5	18.2	5.57–32.7	6
MUFA, mg g^{-1}	34.1^{B,ab}	14.9–49.5	6	49.6^{B,b}	20.9–63.5	5	23.7^{B,a}	5.56–38.8	6
PUFA, mg g^{-1}	26.8^{B}	13.1–47.3	6	33.3	14.4–50.8	5	18.2	7.13–32.8	
n-3 PUFA, mg g^{-1}	23.0^{B}	11.3–41.5	6	27.2^{B}	11.4–43.2	5	15.6	6.37–28.7	6
n-6 PUFA, mg g^{-1}	3.8^{a}	1.77–5.77	6	6.1^{b}	2.91–7.54	5	2.6^{a}	0.75–4.08	6
Herring									
Weight, g	20.7	5.3–42.3	8	19.9	4.3–35.9	8	13.6	2.7–25.4	8
Length, mm	145	100–183	8	144	90–180	8	132	83–170	8

	BPr			BS			GoF		
Variable	Mean	Range	*N*	Mean	Range	*N*	Mean	Range	*N*
CF	0.60^{b}	0.54–0.69	8	0.60^{b}	0.57–0.63	8	0.52^{a}	0.48–0.57	8
Total lipid, %	$5.3^{A,a}$	2.01–7.34	8	$7.4^{A,b}$	4.781–8.803	8	4.0^{a}	2.68–5.57	8
TPP, nmol g^{-1}	0.583	0.424–0.978	8	0.659	0.225–1.411	8	0.436	0.381–0.560	8
TMP, nmol g^{-1}	0.276^{b}	0.138–0.443	8	0.328^{b}	0.120–0.618	8	$0.149^{A,a}$	0.113–0.215	8
THIAM, nmol g^{-1}	4.505	1.851–6.533	8	3.221	2.319–4.312	8	3.791	2.150–5.973	8
TotTh, nmol g^{-1}	5.364	2.538–7.213	8	4.209	3.149–6.341	8	4.375	2.662–6.500	8
TotTh/lipid	1.27	0.41–3.59	8	0.58	0.39–0.77	8	1.15	0.55–1.64	8
TotTh/PUFA	0.43	0.12–1.29	8	0.20	0.11–0.32	8	0.38	0.17–0.54	8
Total carotenoids, mg g^{-1}	0.76^{b}	0.43–1.21	8	0.54^{ab}	0.35–0.69	8	0.43^{a}	0.29–0.75	8
SFA, mg g^{-1}	16.1^{b}	6.31–19.9	8	$22.0^{A,c}$	17.8–25.2	8	12.0^{a}	7.72–17.2	8
MUFA, mg g^{-1}	15.3^{a}	7.29–24.1	8	$24.1^{A,b}$	13.1–31.3	8	$11.8^{A,a}$	8.96–15.7	8
PUFA, mg g^{-1}	16.0^{a}	5.59–20.6	8	22.5^{b}	14.8–31.6	8	11.9^{a}	8.66–17.9	8
n-3 PUFA, mg g^{-1}	12.9^{ab}	4.35–16.8	8	$16.5^{A,b}$	12.1–24.8	8	9.9^{a}	7.39–14.9	8
n-6 PUFA, mg g^{-1}	3.1^{a}	1.24–5.14	8	6.0^{b}	2.71–10.8	8	2.0^{a}	0.91–2.95	8

A different lowercase letter as a superscript to the mean value indicates a significant ($P < 0.05$) difference between the areas, and a different uppercase letter indicates a significant ($P < 0.05$) difference between species within the areas; *N* = number of sprat (1–7 years of age) and herring (1–6 years) pools (each composed of 7–133 individual fish caught in the fall and spring); total lipid contents and the concentrations of FAs were derived from Keinänen et al. [10].

Supplementary Table S4. The effects of species, feeding area, and season on selected parameters of the prey fish of salmon (*Salmo salar*) [sprat (*Sprattus sprattus*) and herring (*Clupea harengus*)] caught in the Baltic Proper (BPr), the Gulf of Finland (GoF), and the Bothnian Sea (BS) in the fall and spring, analyzed by MANOVA, and by LsMeans for significant differences between the means. The parameters were the condition factor (CF) and (on a wet weight basis) the body lipid content, and concentrations of thiamine pyrophosphate (TPP), thiamine monophosphate (TMP), unphosphorylated or free thiamine (THIAM), total thiamine (TotTh) and total carotenoids, and TotTh per lipid and PUFA content, as well as the concentrations of the fatty acid classes.

Variable	MANOVA		Species		Area		Season	
	$F_{9,31}$	$P <$	MANOVA $P <$	LsMeans $P < 0.05$	MANOVA $P <$	LsMeans $P < 0.05$	MANOVA $P <$	LsMeans $P < 0.05$
CF	9.80	0.001			0.001	GoF < BPr, BS	0.001	Spring < fall
Lipid, %	10.0	0.001	0.001	Herring < sprat	0.001	GoF < BPr < BS	0.001	Spring < fall
TPP, nmol g^{-1}	5.15	0.001	ns	ns	0.05	GoF < BPr; BS=BPr, GoF	0.001	Spring < fall
TMP, nmol g^{-1}	3.94	0.01	0.05	Herring < sprat	0.05	GoF < BPr; BS=BPr, GoF	0.01	Spring < fall
THIAM, nmol g^{-1}	0.81	ns	ns	ns	ns	ns	ns	ns
TotTh, nmol g^{-1}	0.56	ns	ns	ns	ns	ns	ns	ns
Total Carotenoids, μg g^{-1}	2.49	0.05	ns	ns	0.01	GoF, BS < BPr; BS=GoF	ns	ns
TotTh/lipid	2.49	0.05	ns	ns	0.1	BS < GoF; BPr=BS, GoF	0.01	Fall < spring
TotTh/PUFA	1.74	ns	ns	ns	ns	ns	0.05	Fall < spring
SFA, mg g^{-1}	7.89	0.001	0.001	Herring < sprat	0.001	GoF < BPr < BS	0.01	Spring < fall
MUFA, mg g^{-1}	9.70	0.001	0.001	Herring < sprat	0.001	GoF, BPr < BS;BPr=GoF	0.001	Spring < fall
PUFA, mg g^{-1}	6.96	0.001	0.001	Herring < sprat	0.001	GoF < BPr < BS	0.001	Spring < fall
n-3 PUFA, mg g^{-1}	6.34	0.001	0.001	Herring < sprat	0.01	GoF < BPr, BS; BPr=BS	0.001	Spring < fall
n-6 PUFA, mg g^{-1}	7.06	0.001	ns	ns	0.001	GoF < BPr < BS	0.01	Spring < fall

The number of sprat and herring pools (each composed of 7–133 individual fish) was 17 and 24 respectively; body lipid contents and the concentrations of FAs were derived from Keinänen et al. [10].
